# Genome‐wide comparative identification and analysis of membrane‐FADS‐like superfamily genes in freshwater economic fishes

**DOI:** 10.1002/2211-5463.13594

**Published:** 2023-03-16

**Authors:** Yuru Zhang, Junmei Zhang, Kedi Gao, Ronghua Lu, Xianglin Cao, Liping Yang, Guoxing Nie

**Affiliations:** ^1^ College of Fisheries Henan Normal University Xinxiang China; ^2^ College of Fisheries, Engineering Technology Research Centre of Henan Province for Aquatic Animal Cultivation Henan Normal University Xinxiang China

**Keywords:** FADS, freshwater economic fishes, gene structure, phylogenetic analysis

## Abstract

Membrane fatty acid desaturase (FADS)‐like superfamily proteins (FADSs) are essential for the synthesis of unsaturated fatty acids (UFAs). Recently, studies on FADS in fishes have mostly focused on marine species, and a comprehensive analysis of the FADS superfamily, including the FADS, stearoyl‐CoA desaturase (SCD), and sphingolipid delta 4‐desaturase (DEGS) families, in freshwater economic fishes is urgently required. To this end, we conducted a thorough analysis of the number, gene/protein structure, chromosomal location, gene linkage map, phylogeny, and expression of the FADS superfamily. We identified 156 *FADSs* genes in the genome of 27 representative species. Notably, *FADS1* and *SCD5* were lost in most freshwater fish and other teleosts. All FADSs proteins contain 4 transmembrane helices and 2–3 amphipathic α‐helices. *FADSs* in the same family are often linked on the same chromosome; moreover, *FADS* and *SCD* or *DEGS* are frequently collocated on the same chromosome. In addition, FADS, SCD, and DEGS family proteins share similar evolutionary patterns. Interestingly, *FADS6*, as a member of the *FADS* family, exhibits a similar gene structure and chromosome location to that of *SCD* family members, which may be the transitional form of *FADS* and *SCD*. This study shed light on the type, structure, and phylogenetic relationship of FADSs in freshwater fishes, offering a new perspective into the functional mechanism analysis of FADSs.

## Graphical abstract

Most genes of the FADS superfamily, which include the FADS, SCD, and DEGS families, typically have 12–11, 6–5, and 3–2 exons and introns, respectively. They share a similar protein architecture (4 transmembrane helices and 2–3 amphipathic a‐helices). The genes/proteins from the same family are usually located on the same chromosome and cluster in a single branch on a phylogenetic tree.

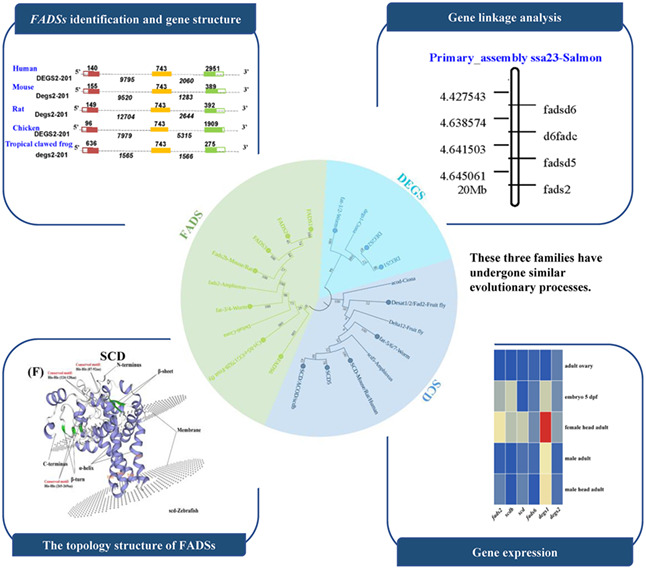

**Abbreviations**
AH: amphipathic a‐helices
ARA: arachidonic acid (20:4n–6)
DEGS: sphingolipid delta 4‐desaturase family
DEGS1: sphingolipid delta 4‐desaturase 1
DEGS2: sphingolipid delta 4‐desaturase 2. The upper‐ and lower‐case letters of gene nomenclature is referring to Ensembl database. Proteins are shown as normal and genes in italics
DHA: docosahexaenoic acid (22:6n–3)
EPA: eicosapentaenoic acid (20:5n–3)
FADS: fatty acid desaturases family
FADS1: fatty acid desaturases 1
FADS2: fatty acid desaturases 2
FADS3: fatty acid desaturases 3
FADS6: fatty acid desaturases 6
*FADSs*
: fatty acid desaturase gene superfamily
FADSs: membrane‐FADS‐like superfamily
LC‐PUFA: long‐chain polyunsaturated fatty acid
MUFA: monounsaturated fatty acids
SCD: stearoyl‐CoA desaturase family
SCD5: stearoyl‐CoA desaturase 5
SCDs: stearoyl‐CoA desaturases
TM: transmembrane helices
UFA: unsaturated fatty acid

Membrane fatty acid desaturase (FADS)‐like superfamily proteins (FADSs) are essential for the synthesis of long‐chain polyunsaturated fatty acid (LC‐PUFA). These enzymes are able to insert carbon–carbon double bonds into the hydrocarbon chains of fatty acids (FAs) at specific sites [1]. According to the HUGO Gene Nomenclature Committee (HGNC) database (https://www.genenames.org/), there are three types of FADSs, namely, the FADS family, the stearoyl‐CoA desaturase (SCD) family, and the sphingolipid delta 4‐desaturase (DEGS) family. The FADS family proteins, composed of an N‐terminal cytochrome b5‐like domain and a C‐terminal multiple membrane‐spanning desaturase portion, generally catalyze the formation of PUFAs from α‐linolenic acid and linoleic acid at the delta‐5 and delta‐6 positions. These enzymes share three highly conserved histidine motifs: RHPGG, LQHDXXH, and HFQHH [2]. SCDs are membrane proteins in the endoplasmic reticulum that catalyze the desaturation of saturated fatty acyl‐CoA substrates at the delta‐9 position, producing essential components of phospholipids, triglycerides, cholesterol esters, and wax esters. They contain three highly conserved histidine motifs: HRLWSH, HRAHH, and HNYHH [3]. DEGS, also known as dihydroceramide desaturases, are key enzymes involved in sphingolipid synthesis, desaturating sphingolipids at the delta‐4 position. They also contain three conserved His motifs of the FADSs superfamily, which are HX_3–4_H, HX_2–3_HH, and (H/Q)X_2–3_HH [4, 5]. On the other hand, based on the catalytic position of different FADSs, they can also be classified as delta 4 (DEGS), delta 5 (FADS1), delta 6 (FADS2), delta 9 (SCD), and other fatty acid desaturases [6, 7].

Fish are the main source of essential FAs, such as arachidonic acid (ARA, 20:4n‐6), eicosapentaenoic acid (EPA, 20:5n‐3), docosahexaenoic acid (DHA, 22:6n‐3), and other LC‐PUFAs for human consumption [8]. Recently, several studies have focused on FADSs in fish. It has been reported that FADS family genes were identified in 40 fish species, including 15 freshwater, 4 diadromous, and 21 marine fish [9]. In marine fishes, the molecular cloning and functional characterization of *FADS* cDNAs involved in the production of EPA and DHA from α‐linolenic acid were conducted with Atlantic cod (*Gadus morhua*) [10], Barramundi (*Lates calcarifer*) [11] and Japanese flounder (*Paralichthys olivaceus*) [12]. Moreover, the mRNA expression of *FADS* has been measured in Atlantic bluefin tuna (*Thunnus thynnus*) [13], Chu's croaker (*Nibea coibor*) [14], and grouper larvae (*Epinephelus coioides*) [15]. In freshwater economic fishes, a specific fatty acid desaturase with two desaturated activities (delta 5 and delta 6) was first identified in zebrafish (*Danio rerio*) [16], followed by the cloning of delta 9 desaturase from grass carp (*Ctenopharyngodon idella*) [7], common carp (*Cyprinus carpio*) [17] and tilapia (*Oreochrom is mossambicus*) [18]. In 2012, Ren et al. [19] cloned two delta 6 desaturase genes from carp. Additionally, a comprehensive review of desaturases involved in PUFA biosynthesis has already been conducted for aquatic invertebrates [20].

Regarding phylogenetic analysis, *fads2* has been discussed in marine economic fishes, such as *Labrus bergylta*, *Sarpa salpa*, *Pegusa lascaris*, *Atherina presbyter*, and aquatic invertebrates [21, 22]. However, existing phylogenetic studies of FADSs have mainly focused on only one FADS family. Moreover, in freshwater fish, there has been limited comprehensive comparative analysis of FADS, SCD, and DEGS, although researchers have been interested in cloning, tissue expression, and function of FADS genes. Therefore, in this study, the FADSs superfamily genes/proteins were analyzed exhaustively from gene types, gene structure, and protein structure to phylogeny. These results can provide a new perspective for exploring the functional mechanism of unknown FADSs members among various fishes.

## Materials and methods

### Organism selection and gene identification

In this work, we chose 27 organisms as representative species. These include common carp (*Cyprinus carpio*), goldfish (*Carassius auratus*), grass carp (*Ctenopharyngodon idellus*), channel catfish (*Ietalurus Punetaus*), yellow catfish (*Tachysurus fulvidraco*), largemouth bass (*Micropterus salmoides*), nile tilapia (*Oreochromis niloticus*), and swamp eel (*Monopterus albus*) as the main freshwater economic fish; Atlantic salmon (*Salmo salar*) and rainbow trout (*Oncorhynchus mykiss*) as the diadromous specie; large yellow croaker (*Larimichthys crocea*), turbot (*Scophthalmus maximus*), fugu (*Takifugu rubripes*), snapper (*Sparus aurata*), cobia (*Rachycentron canadum*), and Atlantic cod (*Gadus morhua*) as the main marine economic fish; human (*Homo sapiens*), mouse (*Mus musculus*), rat (*Rattus norvegicus*), chicken (*Gallus gallus*), tropical clawed frog (*Xenopus tropicalis*), zebrafish (*Danio rerio*), worm (*Caenorhabditis elegans*), and fruit fly (*Drosophila melanogaster*) as key model organisms for the study of lipid metabolism; and urochordata (*Ciona intestinalis*, ciona), cephalochordata (*Branchiostoma belcheri*, amphioxus), and chondrichthian (*Callorhinchus milii*, shark) as useful species for evolutionary analysis.

The HGNC database was used to classify the *FADSs* gene group, and online HGNC comparison of orthology predictions (HCOP) (https://www.genenames.org/tools/hcop/) was employed to identify the orthologues of FADSs in the genomes of humans, mice, rats, chickens, tropical clawed frogs, zebrafish, worms, and fruit flies. Additionally, the orthologues in 19 other organisms were obtained through Ensemble Compare and HomoloGene [23]. To ensure the integrity of FADSs members, NCBI BLASTP and TBLASTN searches with default parameters were conducted, as well as an extensive literature survey to search for previously reported *FADSs* genes. The gene and protein sequences of FADSs in all representative species genomes were downloaded from the NCBI database.

### Gene structure analysis, conserved protein motifs, and topology detection

Referencing the exon–intron organization for the structural diversity of *FADSs* genes in the Ensembl database (release 104), the gene structures, including prominent *FADSs* transcripts, were drawn manually using PowerPoint (PPT).

The identity matrix of FADSs protein sequences was generated by BioEdit [24]. The significant functional motifs of FADSs were identified by the MEME suite [25] (https://meme‐suite.org/meme/doc/meme.html?man_type=web). The 3D structural domains of FADSs proteins were modeled with SWISS‐MODEL [26] (https://swissmodel.expasy.org/), with the protein structure templates of FADS2, SCD, and DEGS1 being rat outer mitochondrial cytochrome b5 (PDBID:1lj0), rat stearoyl‐coenzyme a desaturase 1 (PDBID:4ymk), and human integral membrane stearoyl‐CoA desaturase with substrate (PDBID:4zyo), respectively. The transmembrane topology and signal peptides in the amino acid sequence of zebrafish FADS2, SCD, and DEGS1 were predicted using PolyPhobius (https://phobius.sbc.su.se/poly.html) [27].

### Chromosomal location and gene linkage analysis

For the syntenic analysis of *FADSs* genes, the locations of chromosomes or scaffolds were obtained from Ensembl genome databases (http://asia.ensembl.org/index.html). Subsequently, genetic linkage maps were constructed using mapdraw 2.1 [28].

### Sequence alignment and phylogenetic analysis

DNA and amino acid sequences were aligned by ClustalW with default parameters and then manually inspected. A neighbor‐joining (NJ) phylogenetic tree was built using the Poisson model based on the multiple alignments with 1000 replicates for bootstrap analysis and other default parameters in MEGAX [29]. The reliability of the phylogenetic analysis was verified by constructing a maximum‐likelihood (ML) tree based on the Jones‐Taylor‐Thornton (JTT) model using MEGAX with default parameters. evolview v3 [30] was used to visualize and annotate the NJ tree.

### Expression pattern determination

The gene expression data of zebrafish, tilapia, and salmon were downloaded from the NCBI database. The data were computed from RNA‐seq alignments compared with the most recent RefSeq gene models on the reference genome and then normalized by RPKM (reads per kilobase of transcript per million mapped reads). Heatmaps of gene expression were generated using tbtools (v1.075) [31].

## Results

### Genome‐wide identification of the FADSs superfamily in selected organisms

Referring to the nomenclature and classification of the FADSs in the human genome on the HGNC, we divided the FADSs superfamily into 8 classes, from FADS1 to FADS8. The FADS family comprises FADS1, FADS2, FADS3 and FADS6; the SCD family includes SCD5 (FADS4) and SCDs (FADS5); and the DEGS family consists of DEGS1 (FADS7) and DEGS2 (FADS8). In total, 156 *FADS* genes were identified in the 27 collected organisms (Table 1). The numbers and types of *FADSs* varied among species, with *FADS2* and *FADS6* being the most conserved in the majority of 27 organisms, *FADS1* and *SCD5* being lost in most teleosts, while *DEGS1* and *DEGS2* were found in almost all teleosts.

**Table 1:** Distribution of FADS genes in organisms

FADS^a^ organisms	FADS1 (Δ5)	FADS2 (Δ6)	FADS3 (unknown)	FADS4 (Δ9/SCD5)	FADS5 (Δ9/SCD)	FADS6 (uncertain)	FADS7 (Δ4/DEGS1)	FADS8 (Δ4/DEGS2)	Gene number
Human (*Homo sapiens*)	*FADS1*	*FADS2*	*FADS3*	*SCD5*	*SCD*	*FADS6*	*DEGS1*	*DEGS2*	8
Mouse (*Mus musculus*)	*Fads1*	*Fads2/Fads2b*	*Fads3*	N	*Scd1/Scd2/Scd3/Scd4*	*Fads6*	*Degs1*	*Degs2*	11
Rat (*Rattus norvegicus*)	*Fads1*	*Fads2/Fads2b*	*Fads3*	N	*Scd/Scd2/Scd3/Scd4*	*Fads6*	*Degs1*	*Degs2*	11
Worm (*Caenorhabditis elegans*)	*fat‐3/fat‐4/fat‐5/fat‐6/fat‐7*								5
Chicken (*Gallus gallus*)	*FADS1*	*FADS2*	N	*SCD5*	*SCD*	*FADS6*	*DEGS1*	*DEGS2*	7
Fruit fly (*Drosophila melanogaster*)	*CG17928/Cyt‐b5‐r/Desat1/Desat2/Fad2*								5
Tropical clawed frog (*Xenopus tropicalis*)	*fads1*	*fads2*	N	N	*scd*	*fads6*	*degs1*	*degs2*	6
Zebrafish (*Danio rerio*)	N	*fads2*	N	N	*scd/scdb*	*fads6*	*degs1*	*degs2*	6
Common carp (*Cyprinus carpio*)	N	*fads2a/fads2b*	N	N	*scd*	*fads6*	*degs1*	*degs2*	6
Goldfish (*Carassius auratus*)	N	*fads2*	N	N	*scd/scdb*	*FADS6*	*degs1*	*degs2*	6
Grass carp (*Ctenopharyngodon idella*)	N	*fads2*	N	N	*delta9*	N	GEUQ01062843.1	GEUQ01036533.1	4
Channel catfish (*Ictalurus punctatus*)	N	*fads2*	N	N	*scd/scdb*	*FADS6*	*degs1*	*degs2*	6
Yellow catfish (*Tachysurus fulvidraco*)	N	*fads2*	N	N	*SCD*	*fads6*	*degs1*	*degs2*	5
Nile tilapia (*Oreochromis niloticus*)	N	*Fads2*	N	N	*scd*	*FADS6*	*degs1*	*degs2*	5
Swamp eel (*Monopterus albus*)	N	*fads2*	N	N	*scd*	*fads6*	*degs1*	*degs2*	5
largemouth bass (*Micropterus salmoides*)	N	*fads2*	N	N	*scdb*	*fads6*	*degs1*	*degs2*	5
Rainbow trout (*Oncorhynchus mykiss*)	N	*fads2*	N	*SCD5*	*scdb*	*FADS6*	*degs1*	*degs2*	6
Atlantic salmon (*Salmo salar*)	*fadsd5*	*d6fad_a/d6fad_b/d6fad_c*	N	*SCD5*	*scdb/ACOD*	*FADS6*	*degs1*	*degs2*	10
Large yellow croaker (*Larimichthys crocea*)	N	*fads2*	N	N	*SCD1*	*FADS6*	*degs1*	*degs2*	5
Turbot (*Scophthalmus maximus*)	N	*fads2*	N	N	N	*fads6*	*degs1*	*degs2*	4
Fugu (*Takifugu rubripes*)	N	N	N	N	*scd/scdb*	*FADS6*	*degs1*	*degs2*	5
Snapper (*Sparus aurata*)	N	*FD6D*	N	N	*SCD1a/scdb*	*FADS6*	*degs1*	*degs2*	6
Cobia (*Rachycentron canadum*)	N	*fadsd6*	N	N	N	N	N	N	1
Atlantic cod (*Gadus morhua*)	N	*Fadsd6*	N	N	*scd/scdb*	*FADS6*	*degs1*	*degs2*	6
Shark (*Callorhinchus milii*)	*fads1*	*fads2*	N	*scd5*	*scd*	N	*degs1*	*degs2*	6
Amphioxus (*Branchiostoma belcheri*)	N	*fads2*	N	*scd5*	N	*fads6*	N	N	3
Ciona (*Ciona intestinalis*)	N	*Delta6*	N	N	*acod*	N	*degs1*	N	3

^a^ The upper or lower part of the gene nomenclature refers to the Ensembl database. N—could not be identified. The names in brackets are aliases.

Regarding freshwater economic fish, 6 *fads* genes (*fads2a*, *fads2b*, *scd*, *fads6*, *degs1*, and *degs2*) have been identified in the common carp genome. Goldfish possess similar 6 genes: *fads2*, *scdb*, *scd*, *FADS6*, *degs1*, and *degs2*. In grass carp, there are *fads2*, *delta9*, *degs1*, and *degs2*. Euryhaline fish, such as Atlantic salmon, possess 10 genes: *fadsd5*, *d6fad_a*, *d6fad_b*, *d6fad_c*, *scdb*, *ACOD*, *SCD5*, *FADS6*, *degs1*, and *degs2*. In marine economic fish, Atlantic cod has six *fads* genes (*Fadsd6*, *scd*, *scdb*, *FADS6*, *degs1*, *degs2*). Meanwhile, neither *FADS1* nor *FADS2* is present in the fugu genome. Furthermore, with the updated shark (*C. milii*) genome, we have identified six fads (*fads1*, *fads2*, *scd5*, *scd*, *degs1*, and *degs2*) in cartilaginous fish.

In Table S1, we list the gene ID, protein ID, splice variant number, nucleic acid/protein length, exon/intron numbers, genome sites, and gene annotations of 156 FADSs. The length of the *FADSs* genes varied greatly, from 972 bp (*degs1* in Atlantic cod) to 5556 bp (*fads6* in zebrafish). The protein lengths, however, showed relatively little variation, ranging from 294 (*SCD5* in chicken) to 508 (*fads1* in chicken) amino acids. Generally, each family exhibits similar protein lengths, and the average protein lengths are 444, 342, and 323 aa for FADS, SCD, and DEGS, respectively.

### Structural characterization of *FADSs* genes

**Fig. 1:**
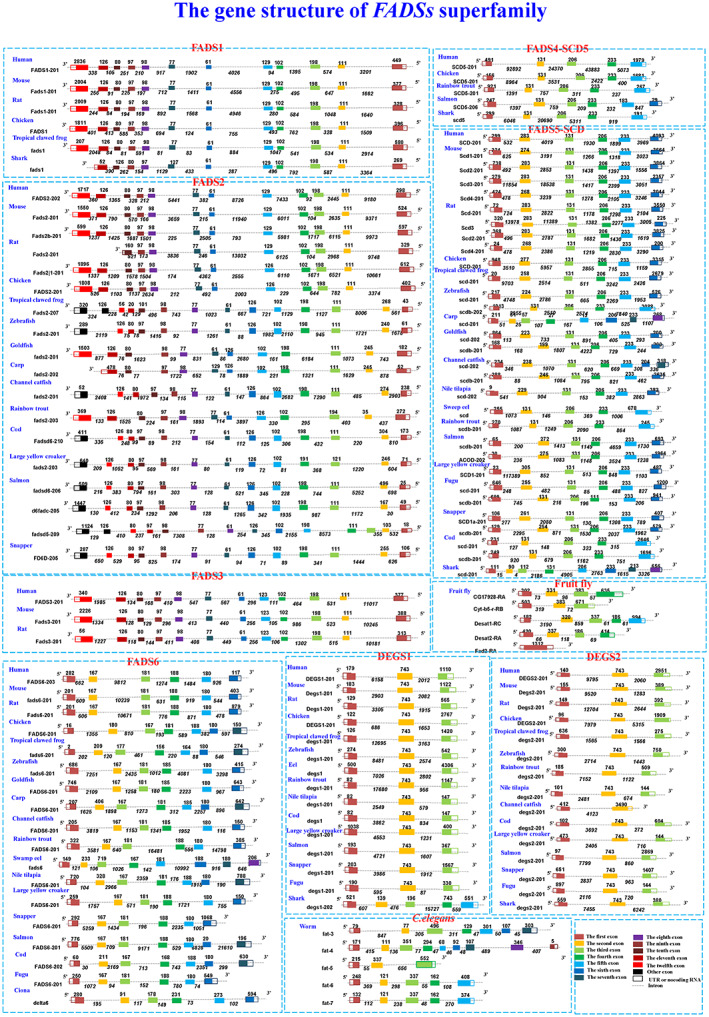
The gene structures of *FADSs* in 27 organisms. Transcripts are presented as boxes (exons) and lines connecting the boxes (introns). Filled boxes represent coding sequences, and unfilled boxes (or portions of boxes) denote UTRs. The length of the exons is indicated on the boxes, and the length of the introns is displayed under the corresponding lines. This view shows representative spliced transcripts for each gene.

To investigate the relationship between structure and function, the gene structures of *FADSs* were analyzed in this study. We downloaded all existing gene structure information of *FADSs* in the Ensembl database and then manually drew classical gene structures without alternative splicing (Fig. 1 and Table S1). Each family displays its own typical structure. Most *FADS1*, *FADS2*, and *FADS3* have 12 exons and 11 introns, with exons 2 to 10 being highly conserved in lengths (111, 198, 102, 129, 61, 77, 98, 97, 80, and 126 bp, respectively). Unlike most FADS family genes, the structure of *FADS6* is more similar to that of *SCD* family genes, which have 6 exons and 5 introns. In addition, some *SCD5* genes show a structure composed of 5 exons and 4 introns, with exons 2–4 exhibiting high conservation (131, 206, and 233 bp, respectively). *DEGS* family genes have 3 exons and 2 introns, with the second exon (743 bp) being highly conserved.

Furthermore, some atypical gene structures are present (Table S2). For instance, 13 exons and 12 introns are present in the *FADS2* of zebrafish, Atlantic cod, channel catfish, *L. crocea*, *S. aurata*, *fadsd6*, and *d6fadc* of Atlantic salmon, respectively; 14 exons and 13 introns are found in the *fadsd5* of Atlantic salmon and *fads2* of Tropical clawed frog; 11 exons and 10 introns are found in the *fads1* of shark and *fads2* of common carp; 8 exons and 7 introns are observed in the *fads2* of Atlantic salmon, *fat*‐*4* of *C. elegans*, *scd* of shark and carp; 5 exons and 4 introns are found in the *degs1* of shark; and 2 exons and 1 intron are present in the *degs2* of channel catfish.

### The conserved His motif and topology structure of FADSs proteins

Although different FADSs family proteins shared limited similarity, the identity of protein sequences varied from 1.1% to 99% (Table S3). Nonetheless, all FADSs have three conserved His‐containing motifs, and homologous proteins with similar functions tend to possess similar architectures. The three conserved motifs, (R/Q)HPGG, LQHDX_2_H, and HFQHH are present in primary structures of most FADS family (FADS1/FADS2/FADS3; Fig. S1A), with the exception of FADS6, which possess the motifs HLAXH, HX3HH, HVEHH (Fig. S1B). Additionally, HRLW(S/A)H, HRXHH, HNXHH (Fig. S2) are conserved in the SCD family, while HX_3_H, HX_2_HH, HXEHH (Fig. S3) are conserved in DEGS family.

**Fig. 2:**
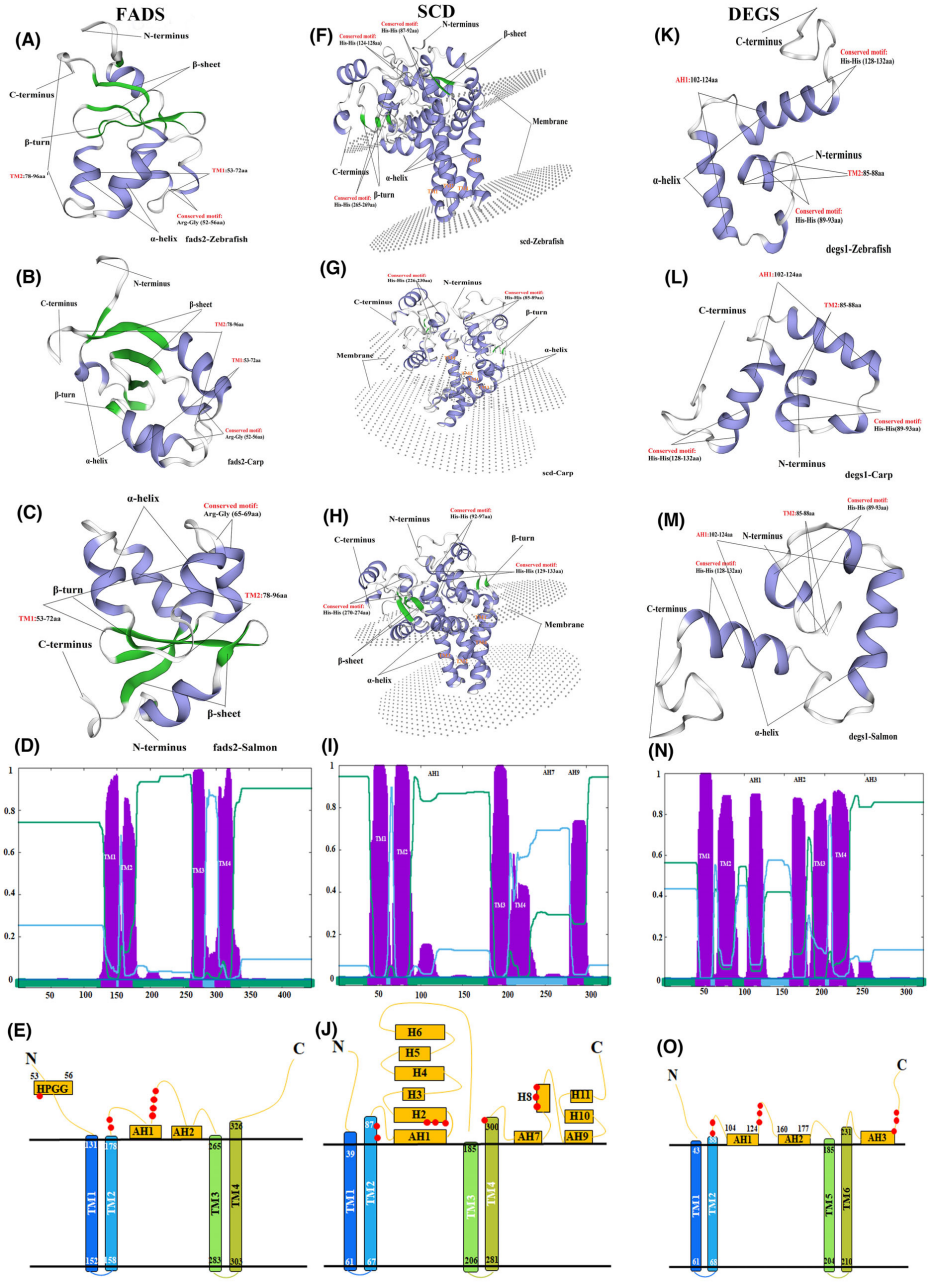
Crystal structure and topology predictions of fads2, scd, and degs1 in zebrafish, common carp, and salmon. The sequences of fads2 mapped with templates in zebrafish (A), common carp (B), and salmon (C) are composed of α‐helices and β‐sheets. The scd of zebrafish (F), common carp (G), and salmon (H) matching the template show 4 TM and 2 AH, and the extracellular domain is made up of α‐helices and β‐sheets. The alignment region of the model template for degs1 of zebrafish (K), common carp (L), and salmon (M) is mainly composed of α‐helices. Plots of fads2 (D), scd (I), and degs1 (N) in zebrafish were generated by Phobius. The probability of residues being located on either side of the membrane is indicated by blue and green, while putative helices are highlighted in purple. E, J, and O show the predicted topological structure model of fads2, scd, and degs1 in zebrafish, respectively. Orange spheres represent conserved histidine residues involved in the coordination of the dimetal centre. This arrangement places all three His‐box motifs (His residues shown as red spheres) on the same side of the membrane.

The crystalline structure of many FADSs proteins has not yet been elucidated. To gain insight into this, we simulated the protein structures of fads2, scd, and degs1 in zebrafish, common carp, and salmon using SWISS‐MODEL. The sequences of fads2 mapped to the template (PDBID:1lj0) show an α‐helix and β‐sheet, which range from 15–98, 15–98, and 29–112 aa in zebrafish, common carp, and salmon, respectively (Fig. 2A–C). The regions that matched the template (PDBID:4ymk) were comparatively integrated for the scd of zebrafish, common carp, and salmon (range: 16–326, 15–286, and 23–330 aa, respectively), comprising the transmembrane helix (TM) and membrane‐bound region composed of amphipathic α‐helix (AH), and the other domain consisting of α‐helix and β‐sheet (Fig. 2F–H). The alignment regions of the model template (PDBID:4zyo) for degs1 of zebrafish, common carp, and salmon were 84–146, 85–145, and 85–146, respectively, which were mainly composed of α‐helices (Fig. 2K–M).

Thereafter, based on the structure of mouse SCD1 [32], we predicted the topological structure of fads2, scd, and degs1 in zebrafish using Plobius. The structures of Fads2 and scd in zebrafish, which contain four helices, were found to be similar to those of SCD1 in mice (Fig. 2D,I). However, degs1 was predicted to have six helices with high probability (Fig. 2N). Careful tracking of the path through the membrane suggested the HX_2_HH motif was placed on the opposite side of the membrane from another His motif, making it impossible to maintain the formation of the metal centre. Therefore, the two helices in the centre of degs1 are likely to be AH‐like AH1 in SCD1. Thus, we propose that degs1 contains 2 AH and 4 TM. We then manually drew the predicted topologies with 4 TM and 2–3 AH of these three proteins (Fig. 2E,J,O).

### Gene linkage map of *FADSs* superfamily

**Fig. 3:**
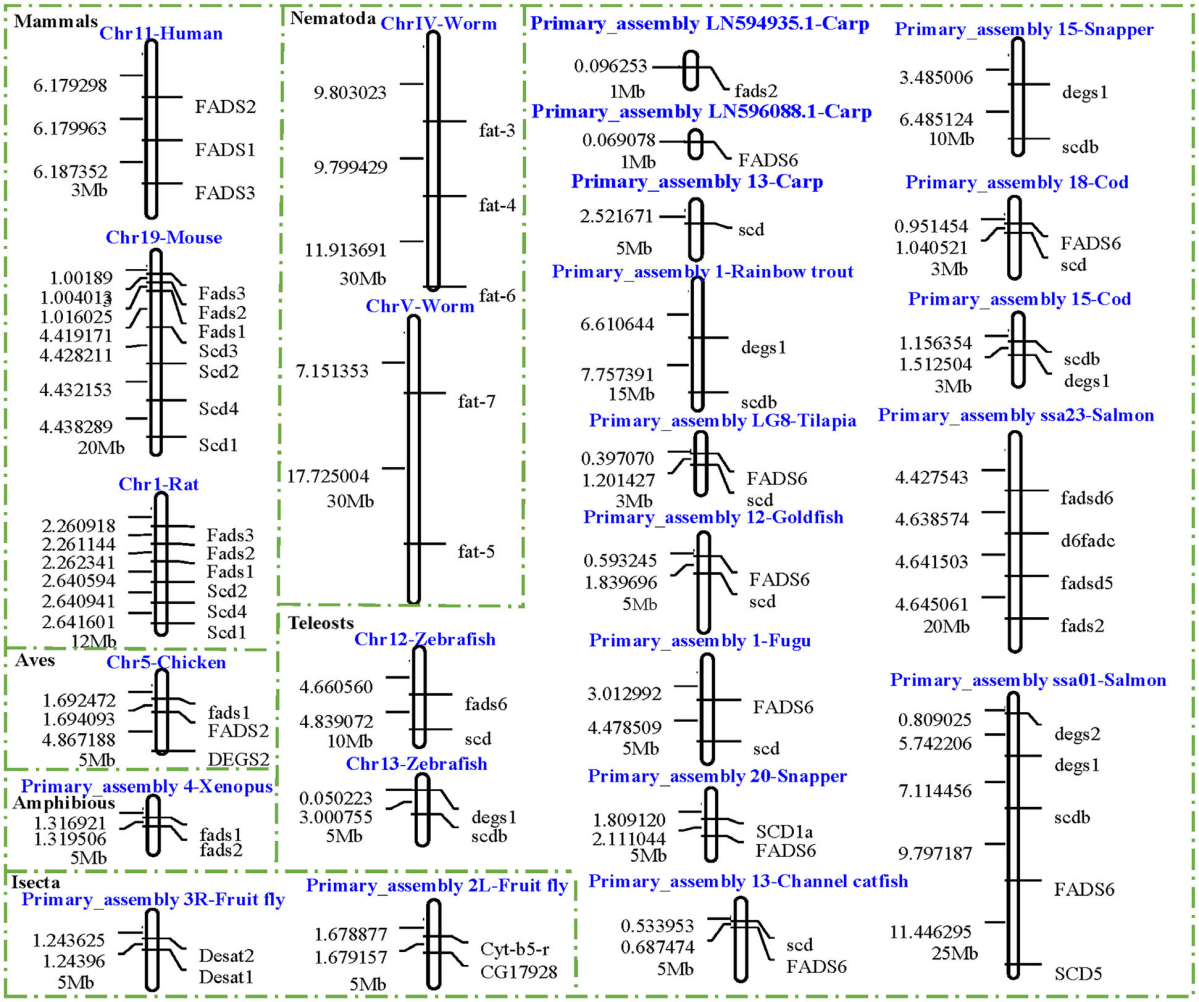
*FADSs* linkage map on chromosomes. The numbers on the left are the FADSs starting points, which were derived from the genome sequences in the Ensembl Genomes database.

Homologous genes are usually linked on the same chromosome; thus, we constructed *FADSs* linkage maps (Fig. 3). In mammals, *FADS1*, *FADS2*, and *FADS3* tend to be localized on the same chromosome and arranged in sequential order on human chromosome 11, mouse chromosome 19 and rat chromosome 1, respectively. The *scd* is also present in chain with *FADS* in rats and mice. Similarly, *fads1* and *fads2* are typically found close together on the same chromosome in chickens and Xenopus. *fat‐3*/*fat‐4*/*fat‐6* and *fat‐5*/*fat‐7* chains are present in nematode. *Fads6*/*scd* and *degs1*/*scdb* are usually situated on the same chromosome in fish. *Degs1*, *degs2*, *scdb*, *FADS6*, and *SCD5* are all located on Primary_assembly ssa01 in Atlantic salmon. Conversely, none of the genes in the three families are located on the same chromosome in common carp.

### Phylogenetic relationship of FADSs

**Fig. 4:**
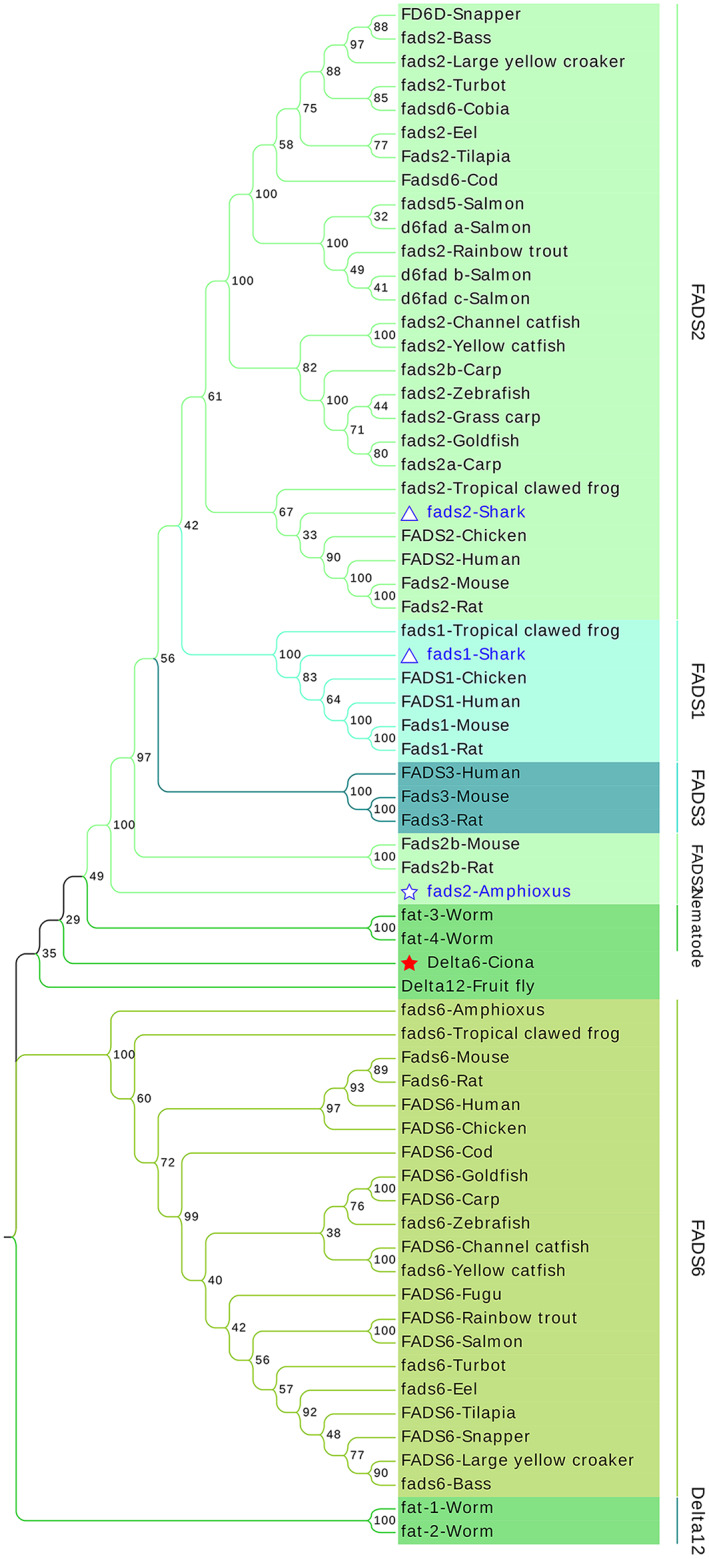
NJ tree showing the evolution of FADS family proteins. The amino acid sequences of FADS proteins were used for phylogenetic analysis based on the NJ method, and the relationships were assessed with bootstrap values (×1000). Solid red star indicates Delta6 of urochordata ciona, hollow blue star indicates fads2 of cephalochordate amphioxus, and hollow blue triangle indicates fads1, fads2 of shark. The FADS family proteins FADS1, FADS2, and FADS3 merge into one clade, while FADS6 is in another clade. The amino acid sequences used in this tree include FADS1 (AAF29378.1), FADS2 (NP_004256.1), FADS3 (NP_068373.1), FADS6 (NP_835229.3) in Human; Fads1 (NP_666206.1), Fads2 (NP_062673.1), Fads2b (NP_001075133.1), Fads3 (NP_068690.3), Fads6 (NP_828874.3) in Mouse; Fads1 (AAG35068.1), Fads2 (BAA75496.1), Fads2b (XP_002729233.1), Fads3 (NP_775160.1), Fads6 (NP_001100534.1) in Rat; fads2 (NP_571720.2), fads6 (XP_003199708.1) in Zebrafish; FADS1 (XP_421052.4), FADS2 (NP_001153900.1), FADS6 (XP_426241.2) in Chicken; fads1 (XP_002943012.2), fads2 (NP_001120262.1), fads6 (XP_012822130.1) in Tropical clawed frog; fads2 (XP_019616342.1), fads6 (XP_019636617.1) in Amphioxus; Delta6 (XP_002131801.1) in Ciona; fads1 (XP_007885635.1), fads2 (XP_007885636.1) in Shark; fads2a (QEN96526.1), fads2b (QEN96527.1), fads6 (XP_018953792.1) in Carp; fads2 (AAS89346.1) in Grass Carp; fads2 (XP_026094349.1), FADS6 (XP_026131844.1) in Goldfish; fads2 (AAK26745.1), FADS6 (XP_021436771.2) in Rainbow trout; fads2 (XP_017341187.1), FADS6 (XP_017338193.1) in Channel catfish; fads2 (XP_027029136.1), fads6 (XP_026989749.1) in Yellow catfish; Fads2 (NP_001266552.1), FADS6 (XP_025765517.1) in Tilapia; fads2 (XP_020475029.1), fads6 (XP_020460422.1) in Eel; fadsd5 (AAL82631.2), d6fad_a (AAU47273.1), d6fad_b (ADB56961.1), d6fad_c (NP_001165251.1), FADS6 (XP_014067950.1) in Salmon; fads2 (XP_038562389.1), fads6 (XP_038591706.1) in Bass; fads2 (NP_001290292.1), FADS6 (XP_010740675.2) in Large yellow croaker; fads2 (XP_035497335.1), fads6 (XP_035468074.1) in Turbot; FADS6 (XP_003961115.3) in Fugu; FD6D (XP_030281225.1), FADS6 (XP_030256488.1) in Snapper; fadsd6 (ACJ65149.1) in Cobia; Fadsd6 (XP_030222643.1), FADS6 (XP_030195240.1) in Cod; fat‐1 (NP_001023560.1), fat‐2 (NP_502560.1), fat‐3 (NP_001255423.1), fat‐4 (NP_001255426.1) in Worm; Delta12 (XP_022211055.2) in Fruit fly.

**Fig. 5:**
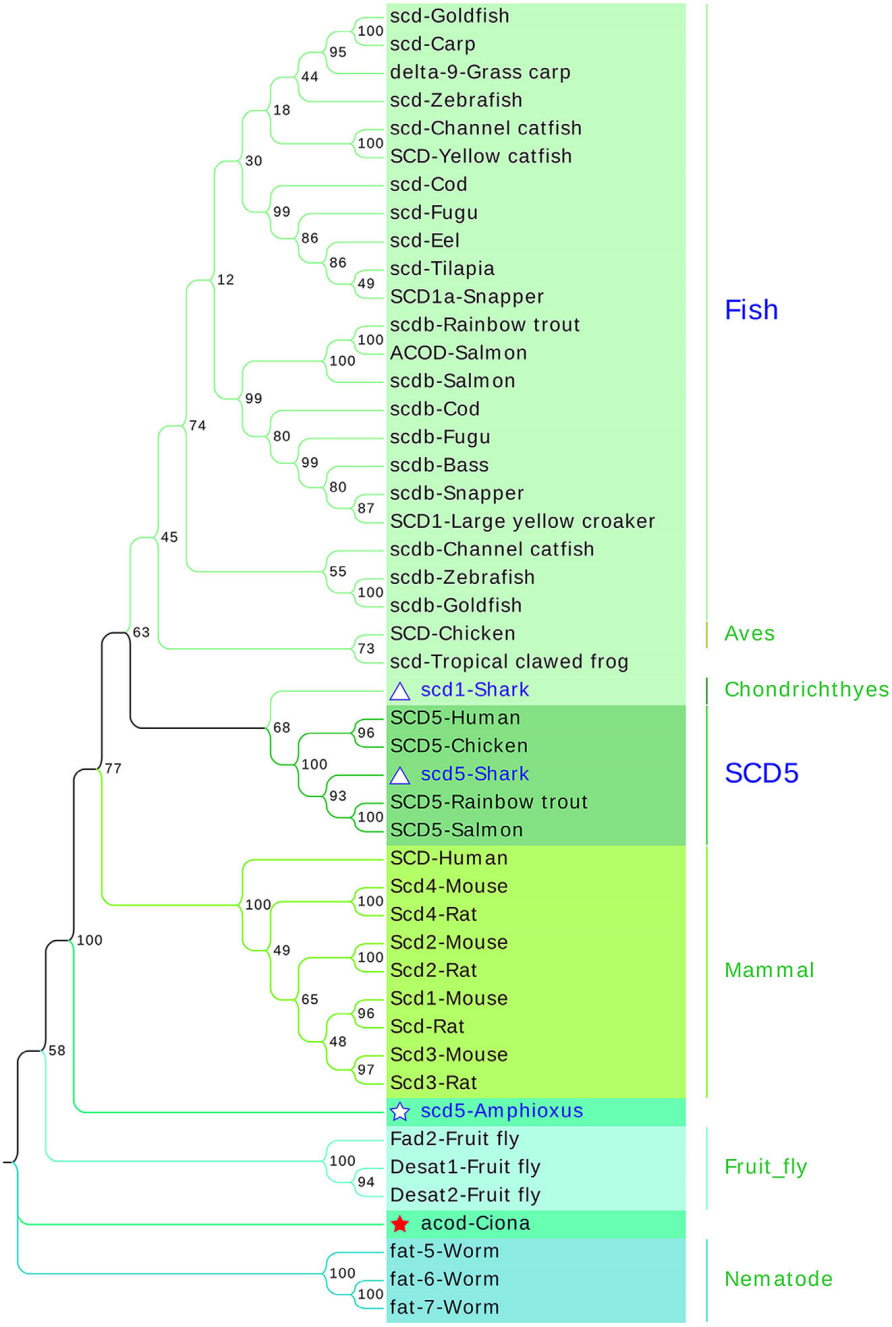
NJ tree showing the evolution of SCD family proteins. In the NJ tree of SCD family members, the SCDs in fruit flies, nematodes, amphioxus, ciona, mammals, chondrichthyes, aves, and fish were branched in different branches. Solid red star indicates acod of urochordata ciona, hollow blue star indicates scd5 of cephalochordate amphioxus, hollow blue triangle indicates scd1, scd5 of shark. The amino acid sequences used in this tree include SCD5 (NP_001032671.2), SCD (NP_005054.3) in Human; Scd1 (NP_033153.2), Scd2 (NP_033154.2), Scd3 (NP_077770.1), Scd4 (NP_899039.2) in Mouse; Scd (NP_631931.2), Scd2 (NP_114029.1), Scd3 (XP_008758709.3), Scd4 (XP_574671.7) in Rat; scdb (NP_001018541.1), scd (NP_942110.2) in Zebrafish; SCD5 (XP_015131966.1), SCD (NP_990221.1) in Chicken; scd (XP_012808654.1) in Tropical clawed frog; scd5 (XP_019635286.1) in Amphioxus; acod (XP_002126680.2) in Ciona; scd5 (XP_007887154.1), scd (XP_007897686.1) in Shark; scd (XP_018961868.1) in Carp; delta9 (CAB53008.1) in Grass Carp; scdb (XP_026117312), scd (XP_026131936) in Goldfish; scdb (XP_021469290.1), SCD5 (XP_021458623.2) in Rainbow trout; scdb (XP_017320070.1), scd (XP_017338153.1) in Channel catfish; SCD (XP_026992014.1) in Yellow catfish; scd (XP_005471439.1) in Tilapia; scd (XP_020460891.1) in Eel; scdb (XP_014061917.1), ACOD (XP_014010587.1), SCD5 (XP_013982607.1) in Salmon; scdb (XP_038593057.1) in Bass; SCD1 (XP_010743587.1) in Large yellow croaker; scdb (NP_001072046.1), scd (NP_001072045.1) in Fugu; scdb (XP_030297377.1), SCD1a (XP_030256516.1) in Snapper; scdb (XP_030234788.1), scd (XP_030196200.1) in Cod; fat‐5 (NP_507482.1), fat‐6 (NP_001255595.1), fat‐7 (NP_504814.1) in Worm; Desat1 (NP_652731.1), Desat2 (NP_650201.1), Fad2 (NP_651966.2) in Fruit fly.

**Fig. 6:**
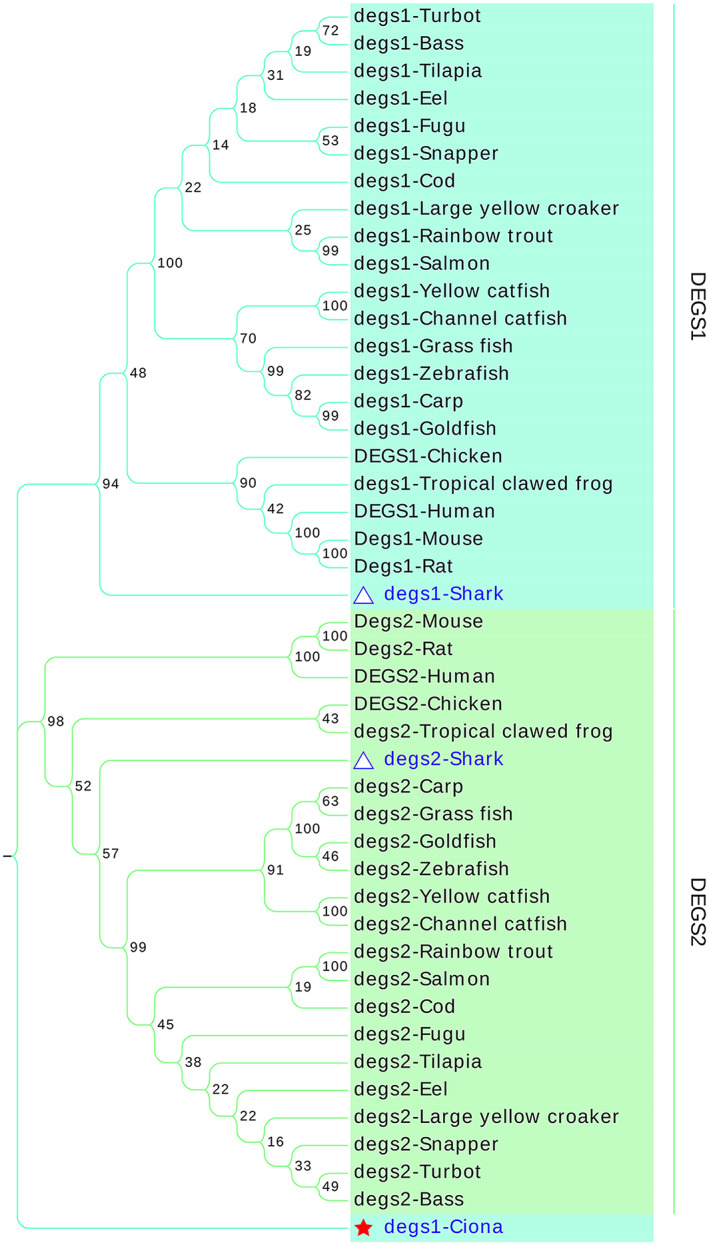
NJ tree showing the evolution of DEGS family proteins. The solid red star indicates degs1 of the cephalochordate amphioxus, the hollow blue triangle indicates degs1, and degs2 indicates shark. The DEGS duplication events predated the urochordata and were then randomly lost in organisms as they evolved. The amino acid sequences used in this tree include DEGS1 (NP_003667.1), DEGS2 (NP_996801.2) in Human; Degs1 (NP_031879.1), Degs2 (NP_081575.2) in Mouse; Degs1 (NP_445775.2), Degs2 (NP_001017457.1) in Rat; degs1 (NP_997865.1), degs2 (NP_001156838.1) in Zebrafish; DEGS1 (NP_001012583.1), DEGS2 (XP_421364.2) in Chicken; degs1 (NP_001007485.1), degs2 (XP_002936627.1) in Tropical clawed frog; degs1 (XP_026690835.1) in Ciona; degs1 (NP_001279205.1), degs2 (XP_007886521.1) in Shark; degs1 (XP_042592224.1), degs2 (XP_042598437.1) in Carp; degs1 (GEUQ01062843.1), degs2 (GEUQ01036533.1) in Grass Carp; degs1 (XP_026080659.1), degs2 (XP_026141964.1) in Goldfish; degs1 (XP_021466074.1), degs2 (XP_021430498.1) in Rainbow trout; degs1 (XP_017317012.1), degs2 (NP_001187333.1) in Channel catfish; degs1 (XP_027030307.1), degs2 (XP_027028203.1) in Yellow catfish; degs1 (XP_003456709.1), degs2 (XP_003453080.1) in Tilapia; degs1 (XP_020463242.1), degs2 (XP_020446493.1) in Eel; degs1 (XP_014058579.1), degs2 (XP_014065160.1) in Salmon; degs1 (XP_038593161.1), degs2 (XP_038585026.1) in Bass; degs1 (XP_010753763.1), degs2 (XP_010732451.1) in Large yellow croaker; degs1 (XP_029688337.1), degs2 (XP_003962526.1) in Fugu; degs1 (XP_030297019.1), degs2 (XP_030247236.1) in Snapper; degs1 (XP_030235813.1), degs2 (XP_030212594.1) in Cod.

Based on the above results, we found that different families shared a limited similarity of protein sequences. Thus, we constructed three separate protein/gene neighbor‐joining (NJ) trees for each family using MEGAX to thoroughly elaborate the evolution of FADSs. The topology of the protein tree was consistent with that of the gene tree. The results of the protein tree were mainly analyzed, while the results of the gene tree are presented in the form of attachments. In the FADS family, FADS1, FADS2, and FADS3 merged into one clade, while FADS6 was in another clade. In the FADS1/FADS2/FADS3 clade, one fads was present in *Branchiostoma belcheri*, which is the orthologues of FADS1/FADS2/FADS3 in vertebrates; conversely, two duplication fads (fads1/fads2) were present in the shark genome (Fig. 4 and Fig. S4). In the SCD family, the SCDs in fruit flies, nematodes, amphioxus, ciona, mammals, chondrichthyes, aves, and fish were branched in different branches; in ciona, there was one SCD that was then duplicated and radiated in vertebrate genomes; however, SCD5 was lost in most teleost fish. Moreover, we found that SCD5 was present in salmon and rainbow trout and clustered with human and cartilaginous fish SCD5 (Fig. 5 and Fig. S5). Similarly, DEGS family proteins underwent a similar evolutionary process to that of the SCD and FADS families (Fig. 6 and Fig. S6).

**Fig. 7:**
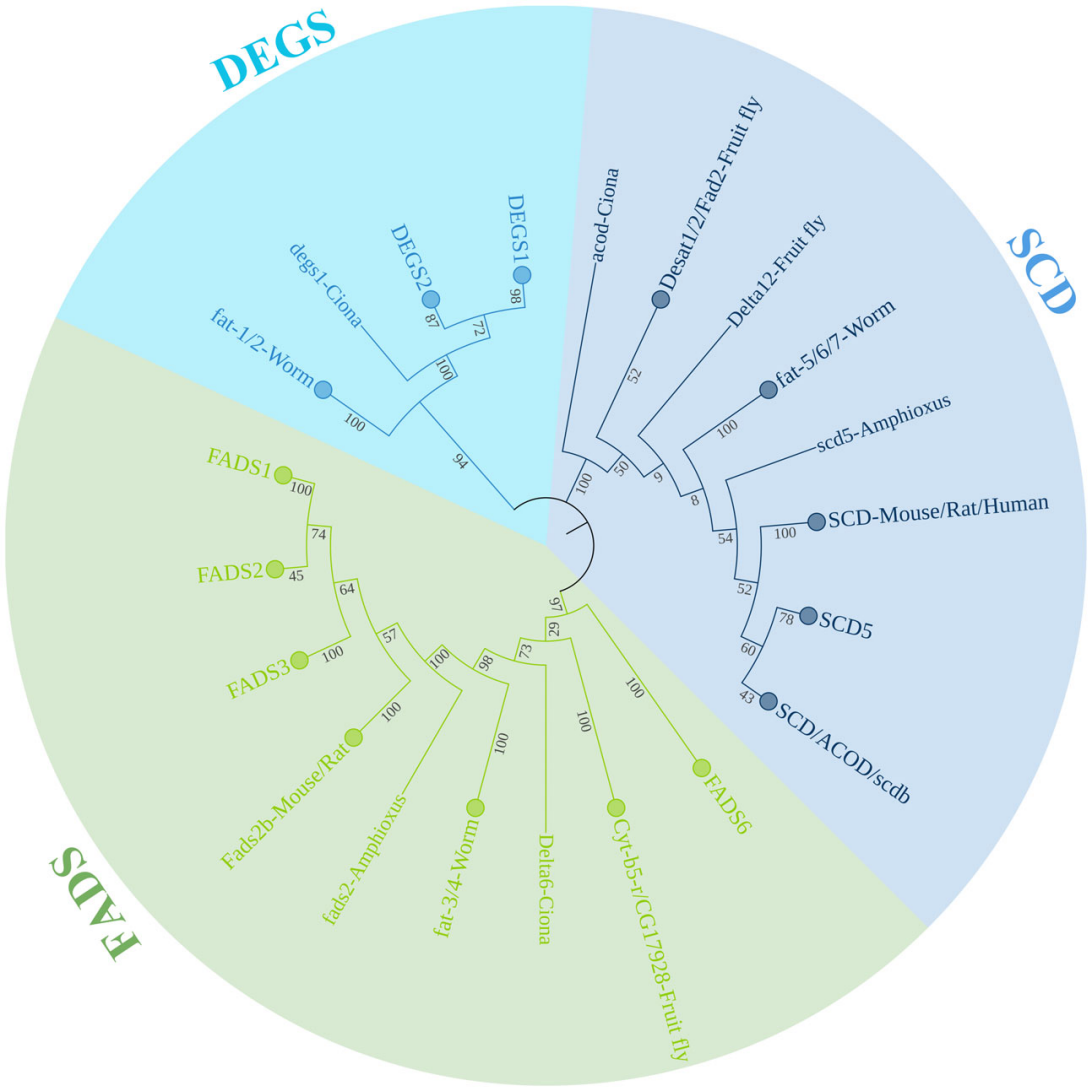
Phylogenetic tree showing the evolution of FADSs. The amino acid sequences of FADSs proteins were used for phylogenetic analysis based on the NJ method, and the relationships were assessed using bootstrap values (×1000). The corresponding accession numbers are detailed in Table S4. To make the phylogenetic tree more legible, we have merged the branches of each family that were clustered together. FADS1/2/3/6 are grouped together as FADS (lightcyan); SCD/ACOD/scdb/SCD5 are grouped into SCD branch (lilac); DEGS1/DEGS2 are clustered into DEGS branch (palegreen); Each branch is highlighted with a unique color. The numbers in the branches represent bootstrap values.

Then, to illustrate the relationship of the FADSs superfamily, we merged all proteins into a single dendrogram (Fig. 7 and Fig. S7). In addition to the above‐investigated sequences, we also selected two Δ12‐related sequences, as LC‐PUFA *de novo* synthesis requires the presence of Δ12 fatty acid desaturase enzymes. We found that FADS, DEGS, and SCD family proteins were divided into three distinct branches in the phylogenetic tree (Fig. 7). The topology of the ML tree of FADSs superfamily proteins (Fig. S8) and genes (Fig. S9) was consistent with that of NJ.

### Gene expression of the *FADSs* superfamily

**Fig. 8:**
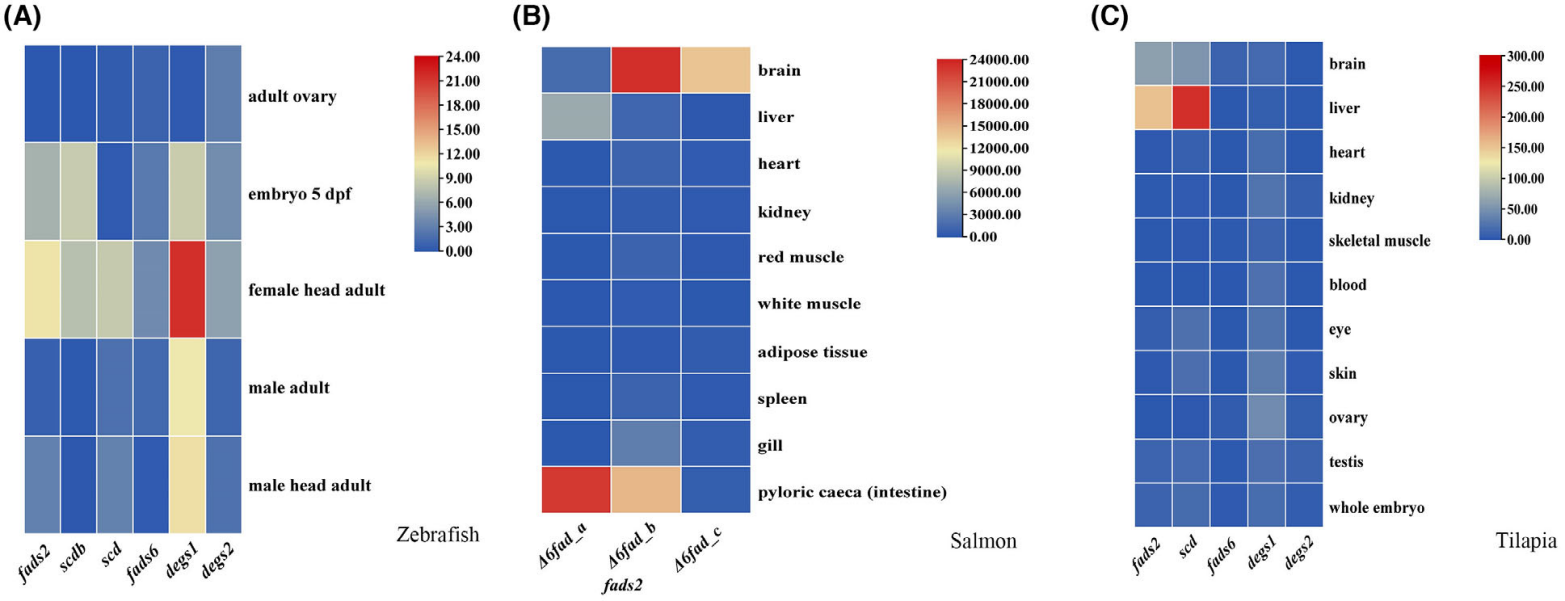
The gene expression of *FADSs* in zebrafish, salmon, and tilapia. The expression of *degs1* was highest in the head of adult female zebrafish (A). *fads2* was highly expressed in the brain and pyloric caeca of Atlantic salmon (B), but the expression of *fads2* in the liver was the highest in tilapia (C). The raw data on the gene expression of three species are provided in Table S5.

Based on data mining from gene expression databases and the literature, gene expression analysis of *FADSs* was performed in zebrafish, Atlantic salmon and tilapia. The results showed that the expression of *degs1* was highest in the heads of adult female zebrafish (Fig. 8A); *fads2* was highly expressed in the brain and pyloric caeca of Atlantic salmon (Fig. 8B), while the expression of *fads2* was the highest in the liver of tilapia (Fig. 8C).

## Discussion

### FADS1 and SCD5 are lost in the majority of fish species

The types, numbers and catalytic activities of *FADSs* are closely related to the biosynthetic capacity of LC‐PUFAs [21]. In this study, we identified the following types of FADSs in 27 representative organisms: FADS (FADS1, FADS2, FADS3, FADS6), SCD (SCDs, SCD5), and DEGS (DEGS1, DEGS2). *FADS1*, *FADS2*, and *FADS3* are found in mammals, and *FADS1* and *FADS2* encode proteins with Δ5 and Δ6 desaturase activities, respectively, while the *FADS3* expression product does not exhibit catalytic activity [33]. By contrast, *FADS1* is lost and *FADS3* is absent in teleosts, while only *FADS2* is conserved in the fish genome. These results are consistent with the previous study [34, 35]. In teleosts, *FADS2* shows multifunctional enzyme activities, including Δ4, Δ5, Δ6, and Δ8 desaturases [16, 36]. This phenomenon confirms that gene loss is a major driving force of functional innovation [37].

A previous study found that due to teleost‐specific genome duplication (3R), the teleost *SCD1* gene repertoire expanded to two copies, but *SCD5* did not [3]. However, in this study, we found that *SCD5* was present in the Atlantic salmon and rainbow trout genomes. Additionally, in freshwater fish, *SCD* was present in the carp and grass carp genomes, yet absent in the channel catfish genomes. This may be due to the improvement of genome sequencing in freshwater fishes. However, the function of the desaturase gene in freshwater fish remains to be studied. In addition, it should be noted that the presence of the above *FADSs* genes is mainly based on the existing research results and fish genome information. The number, type, and function of these genes in specific species must be further studied.

### Exon gain, loss and functional divergence of *FADSs* genes

To characterize the structural information of *FADSs*, we examined 121 gene structures and found that, compared with mammals, most *FADSs* in fish have atypical structures of gene involved in exon gain and loss. For example, in this study, in contrast to the typical *FADS* family gene structure (12 exons and 11 introns), the *FADS2s* of catfish, carp, and large yellow croakers have 13 exons and 11 introns. These structural differences may induce functional divergence of *FADS* genes in fish [38, 39]. Furthermore, the 12th exon of *fads2* is lost in carp, but it still exhibits the ability to synthesize PUFAs [40], suggesting that the 12th exon may not encode the functional site for desaturases interacting with fatty acid substrates. In addition, the high‐quality genomic information of carp is not yet complete, further study is needed to verify the functional mechanism of *fads2*.

Interestingly, as a member of the FADS family, the structure of *FADS6* is similar to that of *SCD* and *SCD5*. We found that the exon of *fads6* has increased in common carp and swamp eel. Similar to the study conducted in *Trachinotus ovatus*, *Fads6* has been shown to possess Δ4 desaturation activity [41]. However, little information on *FADS6* has been reported, and its exact functionality remains to be determined.

### The synteny of *FADSs* genes

Assessing the linkage between family members may provide clues to understanding their evolutionary history [42]. Homologous genes are usually linked on the same chromosome. In this study, we observed that different family genes were frequently present on the same chromosomes. For instance, the linkage of *fads6*/*scd* was found in zebrafish, tilapia, channel catfish, cod, fugu, and goldfish; *degs1*/*scdb* is configured on the same chromosome in zebrafish, rainbow trout, and snapper genome. The synteny of *FADSs* genes may offer a novel insight into the evolution of these three families.

### Three families exhibit similar evolutionary patterns

To understand the evolutionary relationships of the FADSs superfamily, we constructed a phylogenetic tree for individual gene families, as well as phylogenetic trees for the entire superfamily. We found that the three families share a similar evolution pattern. In the FADS1/FADS2/FADS3 clade, only one fads existed in *Branchiostoma belcheri*, while two fads (fads1/fads2) were present in the shark genome. This suggests that the replication and isolation of FADS1 and FADS2 occurred after invertebrate chordate divergence and prior to cartilaginous fishes, with FADS1 being lost in teleost fish. Similarly, as for SCD family members, one SCD in ciona is then duplicated and radiated in vertebrate genomes; however, SCD5 is lost in most teleost fish. Furthermore, DEGS family proteins underwent a similar evolutionary process to that of the SCD and FADS families, with duplication events predated the urochordata and then random losses as the organisms evolved. Thus, these results show that the replication and separation events of the three families (FADS/SCD/DEGS) may have begun in cartilaginous fish.

Interestingly, although the gene structure and protein conserved His motif of FADS6 are similar to those of SCD, it is attached to FADS1/2/3 in the phylogenetic tree but is distant from SCD. Moreover, it has been demonstrated that in humans, the function of FADS6 is comparable to that of SCD [43]. Thus, we speculate that FADS6 may be a transitional form of FADS and SCD, thereby indicating the close kinship of these three families.

Taking into account the similar gene structure, conserved histidine motifs, gene linkage phenomena, and similar evolutionary events, we speculated that all *FADSs* superfamily genes evolved from the same ancestral gene through gene duplication and gene loss; furthermore, these superfamilies subsequently diverged into three families across functional diversification after invertebrate chordate. Therefore, convergent evolution must be considered when viewing the whole superfamily of membrane‐bound desaturases and hydroxylases [4]. However, Δ4 desaturation activity from the FADS and DEGS families is distinct and may be the result of an insertion/deletion event early in the evolution of this superfamily. The fatty acid Δ4‐desaturase and the sphingolipid Δ4‐desaturases most likely have evolved their Δ4‐regioselectivities independently [4]. Furthermore, freshwater and marine fish are likely to cluster together in the evolutionary tree, implying that the family's kinship is not determined by its habitat.

### Specific expression and the possible function of the *FADSs* gene family in the primary LC‐PUFA metabolism pathway

To reveal the expression patterns of the *FADSs* gene superfamily, we investigated gene expression databases and the literature. We found that these genes play different roles in various tissues and sites. For instance, sphingolipids are abundant in the tissues of the central nervous system [44]; therefore, *degs1* exhibits the highest expression in the head of adult female zebrafish. In the fish genome, limited FADSs may possess multiple functions; for example, in Atlantic salmon and tilapia, the expression of *fads2* was inconsistent, likely due to its versatility. However, given the limited data on *FADSs* gene expression in available databases, further research is needed to determine the specific functions of *FADSs* superfamily genes in fish.

**Fig. 9:**
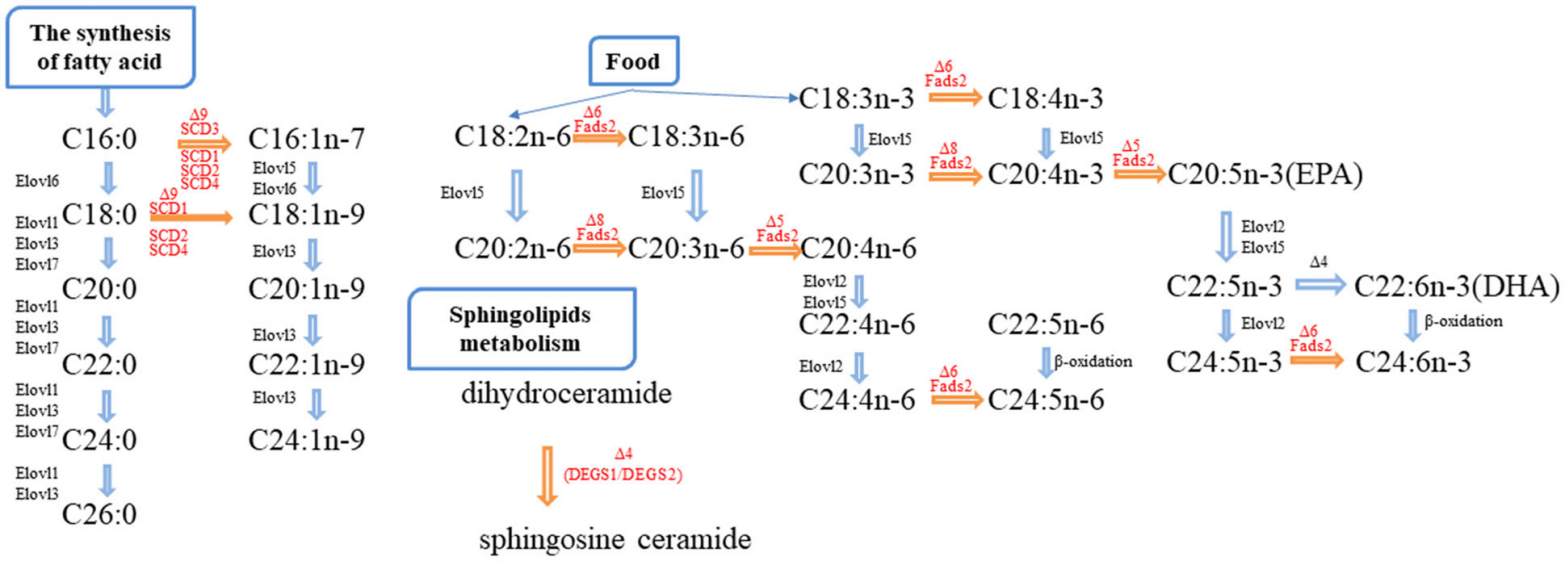
The major metabolic pathways regulated by *FADS* genes in fish.

Functionally, all homologous FADSs perform specific functions at different locations in the PUFA synthesis pathway. According to the functional annotation from PubChem and the literature, we mapped FADSs proteins to the main metabolic pathways in fish (Fig. 9). *FADS2*(Δ5, Δ6, Δ8) primarily catalyze the desaturation of linoleic acid (LA, C18:2n‐6) and α‐linoleic acid (ALA, C18:3n‐3) to form ARA (C20:4n‐6), EPA (C20:5n‐3), and other LC‐PUFAs. *DEGS1*(Δ4) and *DEGS2*(Δ4) promote the synthesis of sphingolipids, mainly catalyzing the formation of sphingosine ceramide from dihydroceramide. *SCD5*(Δ9) and *SCD*(Δ9) are responsible for the desaturation of stearoyl‐ACP (C18:0) (ACP‐acyl carrier protein) to form oleoyl‐ACP (C18:1n‐9).

Fish have a limited ability to endogenously synthesize PUFAs due to the lack of Δ12 and Δ15 desaturases [9]. Instead, they can convert stearic acid to oleic acid by SCD(Δ9). The biosynthesis of LC‐PUFA from ALA usually begins with the desaturation of FADS2(∆6), which then produces ARA and EPA, respectively, through enzyme elongation and subsequent desaturation of FADS1(∆5) [45]. The other pathway, known as the ‘Δ8 pathway’, which involves the elongation of LA and ALA by elongase, followed by desaturation through ∆8 and FADS1(∆5) to generate ARA and EPA, respectively [46]. EPA produced by two different pathways can then be used for DHA synthesis in vertebrates [47]. In general, the most recognized biosynthesis pathway of DHA in vertebrates is the ‘Sprecher pathway’, in which EPA is converted to C24:5n‐3 after two successive elongation enzymes, followed by the desaturation of FADS2(∆6) to C24:6n‐3, and finally produces DHA through partial β‐oxidation. In addition, there is a more direct route from docosapentaenoic acid (DPA, C22:5n‐3) to DHA through the desaturation of ∆4, known as the ‘∆4 pathway’ [47]. We also found the function of another desaturase (DEGS); this enzyme is Δ4 active and acts on sphingolipids, distinct from the Δ4 mentioned above [4].

## Conclusions

To illustrate the relationship between FADSs and the synthesis of UFAs in freshwater economic fish, this study comprehensively conducted a genome‐wide comparative analysis of the *FADSs* gene superfamily, including the FADS, SCD, and DEGS families. A total of 156 *FADSs* genes were identified in 27 organisms. Unlike mammals, *FADS2* with multiple functions is conserved in most freshwater economic fish and other teleosts. Most genes of the *FADS*, *SCD*, and *DEGS* families share 12–11, 6–5, and 3–2 exon–intron typical gene structures, respectively. Moreover, they share a similar protein architecture, consisting of 4 TM and 2–3 AH. The genes/proteins from the same family are usually located on the same chromosome and cluster in a single branch on a NJ tree. Phylogenetic analysis indicates that these three families have undergone similar evolutionary processes.

## Conflict of interest

The authors declare no conflict of interest.

## Author contributions

YZ involved in conceptualization, writing—reviewing and editing, funding acquisition, and project administration; JZ involved in writing—original draft, preparation and investigation of data, data curation, visualization, and Software; KG involved in preparation and investigation of data, and editing; RL involved in data curation and editing; XC involved in data curation and editing; LY involved in data curation and editing; GN involved in supervision and editing.

## Supporting information


**Fig. S1.** FADS family protein multiple sequence alignment and conserved His motif. The three conserved motifs, (R/Q)HPGG, LQHDX2H, and HFQHH are present in primary structures of most FADS family (FADS1/FADS2/FADS3) (A), with the exception of FADS6 (B). In order to clearly demonstrate the His motif, we only present the conserved His and the amino acids that are adjacent to them. The omitted amino acids are indicated by dotted lines. Fig. S2 and Fig. S3 are presented in a similar manner.

**Fig. S2.** Multiple sequence alignment and conserved His motif of SCD family proteins.

**Fig. S3.** The DEGS family protein multiple sequence alignment and conserved His motif.

**Fig. S4.** NJ tree of the *FADS* gene family.

**Fig. S5.** NJ of the *SCD* gene family.

**Fig. S6.** NJ tree of the *DEGS* gene family.

**Fig. S7.** NJ tree of the *FADSs* gene superfamily.

**Fig. S8.** ML tree of FADSs superfamily proteins.

**Fig. S9.** ML tree of *FADSs* superfamily genes.

**Table S1.** *FADSs* gene locations and alternative splicing. In this table, the gene ID, protein ID, splice variant number, RNA/protein length, and exon/intron number, differing genome sites and other gene annotations are listed.

**Table S2.** The atypical structure of protein‐coding genes. Gold shading indicates important freshwater commercial fish.

**Table S3.** Sequence identity matrix of the FADSs. The identities of the protein sequences varied from 1.1% (Fad2 in fruit fly vs. fads2 in grass carp) to 99% (degs2 in grass carp vs. degs2 in common carp).

**Table S4.** List of sequence IDs used for constructing the phylogenetic tree of FADSs superfamily proteins.

**Table S5.** Raw data on gene expression in zebrafish, salmon, and tilapia.

## Data Availability

The data that support the findings of this study are available from the corresponding author upon reasonable request.
