# Supplementary material for: Genome‐wide comparative identification and analysis of membrane‐FADS‐like superfamily genes in freshwater economic fishes
Source: FEBS Open Bio. 2023 Mar 16;13(6):1067–85. doi: 10.1002/2211-5463.13594 (PMC10240347; doi:10.1002/2211-5463.13594)
Supplement: Supplementary file 1 — Fig. S1. FADS family protein multiple sequence alignment and conserved His motif. The three conserved motifs, (R/Q)HPGG, LQHDX2H, and HFQHH are present in primary structures of most FADS family (FADS1/FADS2/FADS3) (A), with the exception of FADS6 (B). In order to clearly demonstrate the His motif, we only present the conserved His and the amino acids that are adjacent to them. The omitted amino acids are indicated by dotted lines. Fig. S2 and Fig. S3 are presented in a similar manner. [file FEB4-13-1067-s013.pdf]

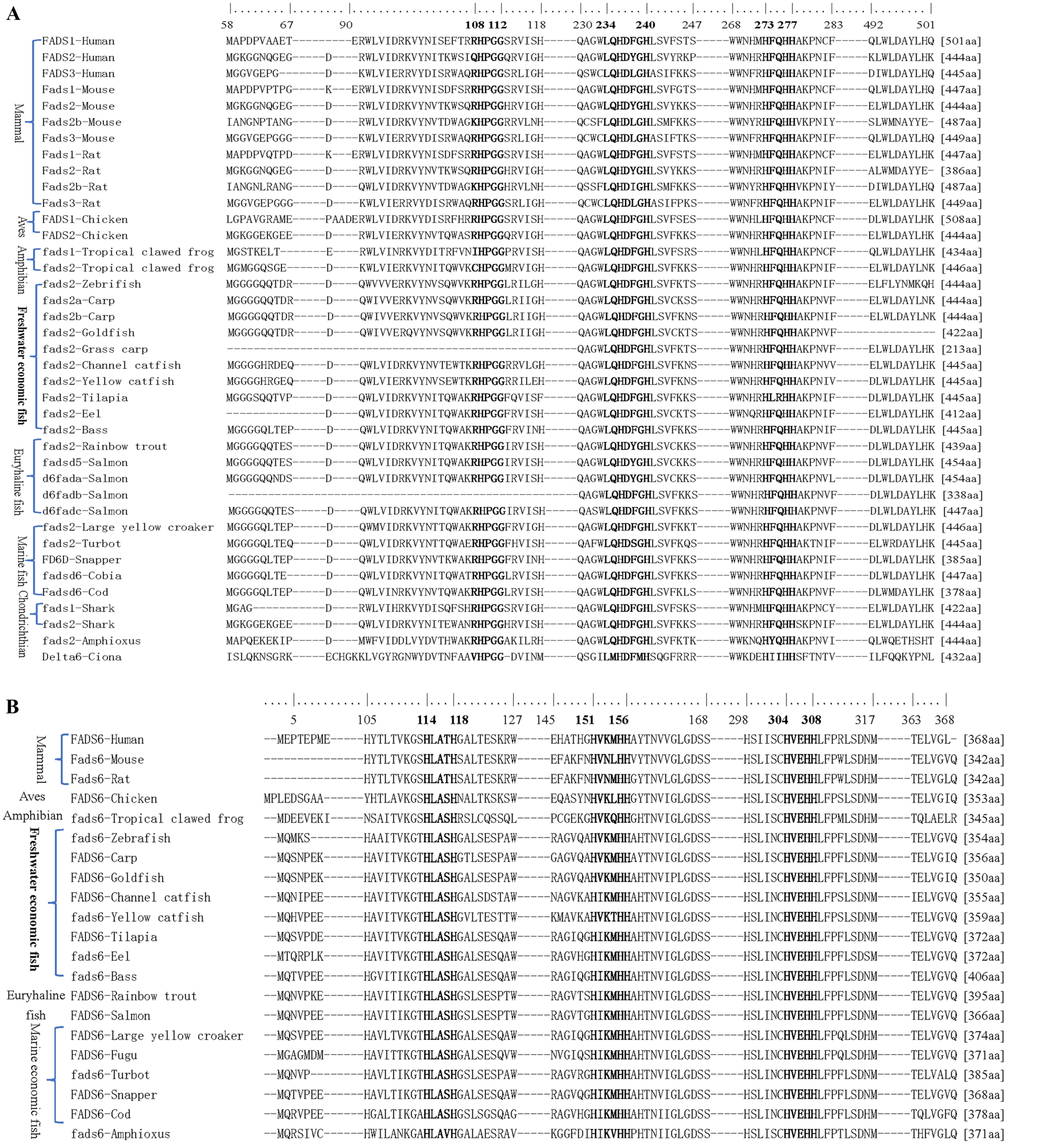
A
58 67 90 108 112 118 230 234 240 247 268 273 277 283 492 501
Mammal
FADS1-Human MAPDPVAAET---------ERWLVIDRKVYNISEFTRRHPGGSRVISH-----QAGWLQHDFGHLSVFSTS-----WWNHMHFQHHAKPNCF-----QLWLDAYLHQ [501aa]
FADS2-Human MGKGGNQGEG-----D----RWLVIDRKVYNITKWSIQHPGGQRVIGH-----QAGWLQHDYGHLSVYRKP-----WWNHRHFQHHAKPNIF-----KLWLDAYLHK [444aa]
FADS3-Human MGGVGEPG-------D----KWLVIERRVYDISRWAQRHPGGSRLIGH-----QSWCLQHDLGHASIFKKS-----WWNFRHFQHHAKPNIF-----DIWLDAYLHQ [445aa]
Fads1-Mouse MAPDPVPTPG-----K---ERWLVIDRKVYNISDFSRRHPGGSRVISH-----QAGWLQHDFGHLSVFGTS-----WWNHMHFQHHAKPNCF-----QLWLDAYLHQ [447aa]
Fads2-Mouse MGKGGNQGEG-----D----RWLVIDRKVYNVTKWSQRHPGGHRVIGH-----QAGWLQHDYGHLSVYKKS-----WWNHRHFQHHAKPNIF-----ELWLDAYLHK [444aa]
Fads2b-Mouse IANGNPTANG-----D----RWLVIDRKVYNVTDWAGKHPGGRRVLNH-----QCSFLQHDLGHLSMFKKS-----WWNYRHFQHHVKPNIY-----SLWMNAYYE- [487aa]
Fads3-Mouse MGGVGEPGGG-----D----KWLVIERRVYDISRWAQRHPGGSRLIGH-----QCWCLQHDLGHASIFTKS-----WWNFRHFQHHAKPNIF-----QLWLDAYLHQ [449aa]
Fads1-Rat MAPDPVQTPD-----K---ERWLVIDRKVYNISDFSRRHPGGSRVISH-----QAGWLQHDFGHLSVFSTS-----WWNHMHFQHHAKPNCF-----ELWLDAYLHK [447aa]
Fads2-Rat MGKGGNQGEG-----D----RWLVIDRKVYNVTKWSQRHPGGHRVIGH-----QAGWLQHDYGHLSVYKKS-----WWNHRHFQHHAKPNIF-----ALWMDAYYE- [386aa]
Fads2b-Rat IANGNLRANG-----D----QWLVIDRKVYNVTDWAGKHPGGHRVLNH-----QSSFLQHDIGHLSMFKKS-----WWNYRHFQHHVKPNIY-----DIWLDAYLHQ [487aa]
Fads3-Rat MGGVGEPGGG-----D----KWLVIERRVYDISRWAQRHPGGSRLIGH-----QCWCLQHDLGHASIFPKS-----WWNFRHFQHHAKPNIF-----ELWLDAYLHK [449aa]
Aves
FADS1-Chicken LGPAVGRAME-----PAADERWLVIDRKVYDISRFHRRHPGGSRVISH-----QAGWLQHDFGHLSVFSES-----WWNHLHFQHHAKPNCF-----DLWLDAYLHK [508aa]
FADS2-Chicken MGKGGEKGEE-----D----RWLVIERKVYNVTQWASRHPGGQRVIGH-----QAGWLQHDFGHLSVFKKS-----WWNHRHFQHHAKPNIF-----ELWLDAYLHK [444aa]
Amphibian
fads1-Tropical clawed frog MGSTKELT-------E----RWLVINRKVYDITRFVNIHPGGPRVISH-----QAGWLQHDFGHLSVFSRS-----WWNHLHFQHHAKPNCF-----QLWLDAYLHK [434aa]
fads2-Tropical clawed frog MGMGGQSGE------D----KWLVIERKVYNITQWVKCHPGGMRVIGH-----QAGWLQHDFGHLSVFKKS-----WWNHRHFQHHAKPNIF-----ELWLDAYLNK [446aa]
Freshwater economic fish
fads2-Zebrifish MGGGGQQTDR-----D----QWVVVERKVYNVSQWVKRHPGGLRILGH-----QAGWLQHDFGHLSVFKTS-----WWNHRHFQHHAKPNIF-----ELFLYNMKQH [444aa]
fads2a-Carp MGGGGQQTDR-----D----QWIVVERKVYNVSQWVKRHPGGLRIIGH-----QAGWLQHDFGHLSVCKSS-----WWNHRHFQHHAKPNVF-----ELWLDAYLNK [444aa]
fads2b-Carp MGGGGQQTDR-----D----QWIVVERKVYNVSQWVKRHPGGLRIIGH-----QAGWLQHDFGHLSVFKNS-----WWNHRHFQHHAKPNIF-----ELWLDAYLNK [444aa]
fads2-Goldfish MGGGGQQTDR-----D----QWIVVERQVYNVSQWVKRHPGGLRIIGH-----QAGWLQHDFGHLSVCKTS-----WWNHRHFQHHAKPNVF--------------- [422aa]
fads2-Grass carp --QAGWLQHDFGHLSVFKTS-----WWNHRHFQHHAKPNVF-----ELWLDAYLHK [213aa]
fads2-Channel catfish MGGGGHRDEQ-----D----QWLVIDRKVYNVTEWTKRHPGGRRVLGH-----QAGWLQHDFGHLSVFKNS-----WWNHRHFQHHAKPNVV-----ELWLDAYLHK [445aa]
fads2-Yellow catfish MGGGGHRGEQ-----D----QWLVIERKVYNVSEWTKRHPGGRRILEH-----QAGWLQHDFGHLSVFKNS-----WWNHRHFQHHAKPNIV-----DLWLDAYLHK [445aa]
Fads2-Tilapia MGGGSQQTVP-----D----QWLVIDRKVYNITQWAKRHPGGFQVISF-----QAGWLQHDFGHLSVFKKS-----WWNHRHLRHHAKPNIF-----DLWLDAYLHK [445aa]
fads2-Eel ---------------D----QWLVIDRKVYNITQWAKRHPGGFRVISH-----QAGWLQHDFGHLSVCKTS-----WWNQRHFQHHAKPNIF-----ELWLDAYLHK [412aa]
fads2-Bass MGGGGQLTEP-----D----QWLVIDRKVYNITQWAKRHPGGFRVINH-----QAGWLQHDFGHLSVFKKS-----WWNHRHFQHHAKPNIF-----DLWLDAYLHK [445aa]
Euryhaline fish
fads2-Rainbow trout MGGGGQQTES-----D----QWLVIDRKVYNITQWAKRHPGGIRVISH-----QAGWLQHDYGHLSVCKKS-----WWNHRHFQHHAKPNVF-----DLWLDAYLHK [439aa]
fadsd5-Salmon MGGGGQQTES-----D----QWLVIDRKVYNITQWAKRHPGGIRVISH-----QAGWLQHDYGHLSVCKKS-----WWNHRHFQHHAKPNVF-----DLWLDAYLHK [454aa]
d6fada-Salmon MGGGGQQNDS-----D----QWLVIDRKVYNITQWAKRHPGGIRVISH-----QAGWLQHDYGHLSVCKKS-----WWNHRHFQHHAKPNVL-----DLWLDAYLHK [454aa]
d6fadb-Salmon --QAGWLQHDFGHLSVFKKS-----WWNHRHFQHHAKPNVF-----DLWLDAYLHK [338aa]
d6fadc-Salmon MGGGGQQTES-----D----QWLVIDRKVYNITQWAKRHPGGIRVISH-----QASWLQHDFGHLSVFKKS-----WWNHRHFQHHAKPNVF-----DLWLDAYLHK [447aa]
Marine fish
fads2-Large yellow croaker MGGGGQLTEP-----D----QWMVIDRKVYNTTQWAKRHPGGFRVIGH-----QAGWLQHDFGHLSVFKKT-----WWNHRHFQHHAKPNVF-----DLWLDAYLHK [446aa]
fads2-Turbot MGGGGQLTEQ-----D----QWLVIDRKVYNTTQWAERHPGGFHVISH-----QAFWLQHDSGHLSVFKQS-----WWNHRHFQHHAKTNIF-----ELWRDAYLHK [445aa]
FD6D-Snapper MGGGGQLTEP-----D----QWLVIDRKVYNVTKWAKRHPGGFRVINH-----QAGWLQHDFGHLSVFKKS-----WWNHRHFQHHAKPNIF-----DLWLDAYLHK [385aa]
fadsd6-Cobia MGGGGQLTE------D----QWLVIDRKVYNITQWATRHPGGLRVISH-----QAGWLQHDFGHLSVFKKS-----WWNHRHFQHHAKPNIF-----DLWLDAYLHK [447aa]
Fadsd6-Cod MGGGGQLTEP-----D----QWLVINRKVYNVTQWAKRHPGGLRVISH-----QAGWLQHDFGHLSVFKLS-----WWNHRHFQHHAKPNVF-----DLWMDAYLHK [378aa]
Chondrichthian
fads1-Shark MGAG-----------D----RWLVIHRKVYDISQFSHRHPGGSRVIGH-----QAGWLQHDFGHLSVFSKS-----WWNHMHFQHHAKPNCY-----ELWLDAYLHK [422aa]
fads2-Shark MGKGGEKGEE-----D----QWLVIDRKVYNITEWANRHPGGHRVISH-----QAGWLQHDFGHLSVFKKS-----WWNHRHFQHHSKPNIF-----ELWLDAYLHK [444aa]
fads2-Amphioxus MAPQEKEKIP-----D----MWFVIDDLVYDVTHWAKRHPGGAKILRH-----QAGWLQHDFGHLSVFKTK-----WWKNQHHYQHHAKPNVI-----QLWQETHSHT [444aa]
Delta6-Ciona ISLQKNSGRK-----ECHGKKLVGYRGNWYDVTNFAAVHPGG-DVINM-----QSGILMHDFMHSQGFRRR-----WWKDEHIIHHSFTNTV-----ILFQQKYPNL [432aa]
B
5 105 114 118 127 145 151 156 168 298 304 308 317 363 368
Mammal
FADS6-Human --MEPTEPME-----HYTLTVKGSHLATHGALTESKRW-----EHATHGHVKMHHAYTNVVGLGDSS-----HSIISCHVEHHLFPRLSDNM-----TELVGL- [368aa]
Fads6-Mouse ---------------HYTLTVKGSHLATHSALTESKRW-----EFAKFNHVNLHHVYTNVVGLGDSS-----HSLISCHVEHHLFPWLSDHM-----TELVGVQ [342aa]
Fads6-Rat ---------------HYTLTVKGSHLATHSALTESKRW-----EFAKFNHVNMHHGYTNVLGLGDSS-----HSLISCHVEHHLFPWLSDHM-----TELVGLQ [342aa]
Aves
FADS6-Chicken MPLEDSGAA------YHTLAVKGSHLASHNALTKSKSW-----EQASYNHVKLHHGYTNVIGLGDSS-----HSLISCHVEHHLFPSLSDNM-----TELVGIQ [353aa]
Amphibian
fads6-Tropical clawed frog --MDEEVEKI-----NSAITVKGSHLASHRSLCQSSQL-----PCGEKGHVKQHHGHTNVIGLGDSS-----HSLISCHVEHHLFPMLSDHM-----TQLAELR [345aa]
Freshwater economic fish
fads6-Zebrafish --MQMKS--------HAAITVKGTHLASHGALSESPAW-----RAGVQAHVKMHHAHTNVIGLGDSS-----HSLINCHVEHHLFPFLSDNM-----TELVGVQ [354aa]
FADS6-Carp --MQSNPEK------HAVITVKGTHLASHGTLSESPAW-----GAGVQAHVKMHHAYTNVIGLGDSS-----HSLISCHVEHHLFPFLSDNM-----TELVGIQ [356aa]
FADS6-Goldfish --MQSNPEK------HAVITVKGTHLASHGALSESPAW-----RAGVQAHVKMHHAHTNVIPLGDSS-----HSLISCHVEHHLFPFLSDNM-----TELVGIQ [350aa]
FADS6-Channel catfish --MQNIPEE------HAVITVKGTHLASHGALSDSTAW-----NAGVKAHIKMHHAHTNVIGLGDSS-----HSLINCHVEHHLFPSLSDNM-----IELVGVQ [355aa]
fads6-Yellow catfish --MQHVPEE------HAVITVKGTHLASHGVLTESTTW-----KMAVKAHVKTHHAHTNVIGLGDSS-----HSLINCHVEHHLFPSLSDNM-----TELVGVQ [359aa]
FADS6-Tilapia --MQSVPDE------HAVITVKGTHLASHGALSESQAW-----RAGIQGHIKMHHAHTNVIGLGDSS-----HSLINCHVEHHLFPFLSDNM-----TELVGVQ [372aa]
fads6-Eel --MTQRPLK------HAVITVKGTHLASHGALSESQAW-----RAGVHGHIKMHHAHTNVIGLGDSS-----HSLINCHVEHHLFPFLSDSM-----TELVGVQ [372aa]
fads6-Bass --MQTVPEE------HGVITIKGTHLASHGALSESQAW-----RAGIQGHIKMHHAHTNVIGLGDSS-----HSLINCHVEHHLFPFLSDNM-----TELVGVQ [406aa]
Euryhaline fish
FADS6-Rainbow trout --MQNVPKE------HAVITIKGTHLASHGSLSESPTW-----RAGVTSHIKMHHAHTNVIGLGDSS-----HSLINCHVEHHLFPFLSDNM-----TELVGVQ [395aa]
FADS6-Salmon --MQNVPEE------HAVITIKGTHLASHGSLSESPTW-----RAGVTGHIKMHHAHTNVIGLGDSS-----HSLINCHVEHHLFPFLSDNM-----TELVGVQ [366aa]
Marine economic fish
FADS6-Large yellow croaker --MQSVPEE------HAVLTVKGTHLASHGALSESQAW-----RAGIQGHIKMHHAHTNVIGLGDSS-----HSLINCHVEHHLFPQLSDHM-----TELVGVQ [374aa]
FADS6-Fugu --MGAGMDM------HAVITTKGTHLASHGALSESQVW-----NVGIQSHIKMHHAHTNVIGLGDSS-----HSLINCHVEHHLFPQLSDHM-----TELVGVQ [371aa]
fads6-Turbot --MQNVP--------HAVLTIKGTHLASHGSLSESPAW-----RAGVRGHIKMHHAHTNVIGLGDSS-----HSLINCHVEHHLFPFLSDNM-----TELVALQ [385aa]
FADS6-Snapper --MQTVPEE------HAVLTIKGTHLASHGALSESQAW-----RAGVQGHIKMHHAHTNVIGLGDSS-----HSLINCHVEHHLFPFLSDNM-----TELVGVQ [368aa]
FADS6-Cod --MQRVPEE------HGALTIKGAHLASHGSLSGSQAG-----RAGVHGHIKMHHAHTNIIGLGDSS-----HSLINCHVEHHLFPFLSDHM-----TQLVGFQ [378aa]
fads6-Amphioxus --MQRSIVC------HWILANKGAHLAVHGALAESRAV-----KGGFDIHIKVHHPHTNIIGLGDSS-----HSLISCHVEHHLFPTLSDNM-----THFVGLQ [371aa]
