# Supplementary material for: Genome‐wide comparative identification and analysis of membrane‐FADS‐like superfamily genes in freshwater economic fishes
Source: FEBS Open Bio. 2023 Mar 16;13(6):1067–85. doi: 10.1002/2211-5463.13594 (PMC10240347; doi:10.1002/2211-5463.13594)
Supplement: Supplementary file 2 — Fig. S2. Multiple sequence alignment and conserved His motif of SCD family proteins. [file FEB4-13-1067-s002.pdf]

Group	Sequence	66 … 75 … 114 … 120 … 125 … 131 … 152 … 157 161 … 167 … 293 … 298 302 … 307 … 350 … 359	Length
Mammal	SCD-Human	SPKVEYVWRN-----GITAGAHRLWSHRSYKAR-----EWARDHRAHHKFSETH-----VGEGFHNYHHSFPYD-----RTGDGNYKSG	[359aa]
Mammal	SCD5-Human	---QNIVWRN-----GVTAGAHRLWSHRSYRAK-----EWSRDHRAHHKYSETD-----IGEGFHNYHHTFPFD-----RTGDSSA---	[330aa]
Mammal	Scd1-Mouse	PPKLEYVWRN-----GITAGAHRLWSHRTYKAR-----EWARDHRAHHKFSETH-----VGEGFHNYHHTFPFD-----RTGDGSHKSS	[355aa]
Mammal	Scd2-Mouse	PPKLEYVWRN-----GITAGAHRLWSHRTYKAR-----EWARDHRAHHKFSETH-----VGEGFHNYHHAFPYD-----RTGDGSCKSG	[358aa]
Mammal	Scd3-Mouse	PPKLEYVWRN-----GIGAGVHRLWSHRTYKAR-----EWARDHRAHHKFSETH-----MGEGFHNYHHAFPYD-----RTGDGSHKSG	[359aa]
Mammal	Scd4-Mouse	PPKLEYVWRN-----GITAGAHRLWSHRTYKAR-----EWARDHRAHHKFSETH-----LGEGFHNYHHTFPYD-----RTGDGSHKSS	[353aa]
Mammal	Scd-Rat	PPKLEYVWRN-----GITAGAHRLWSHRTYKAR-----EWARDHRAHHKFSETH-----VGEGFHNYHHAFPYD-----RTGDGSHKSS	[358aa]
Mammal	Scd2-Rat	PPKLEYVWRN-----GITAGAHRLWSHRTYKAR-----EWARDHRAHHKFSETH-----VGEGFHNYHHAFPYD-----RTGEESCKSG	[358aa]
Mammal	Scd3-Rat	PPKLEYVWRN-----GIGAGVHRLWSHRTYKAR-----EWARDHRAHHKFTETH-----LGEGFHNYHHAFPYD-----RTGDGSHKSG	[359aa]
Mammal	Scd4-Rat	PPKLEYVWRN-----GITAGAHRLWSHRTYKAR-----EWARDHRAHHKFSETH-----LGEGFHNYHHAFPYD-----RTGDGSHKSS	[353aa]
Aves	SCD-Chicken	KPPLRYVWRN-----GITAGSHRLWSHRSYKAT-----EWARDHRVHHKFSETH-----LGEGFHNYHHTFPYD-----RTGDGSHKSG	[582aa]
Aves	SCD5-Chicken	---------------GVTAGAHRLWSHRSYKAK-----EWSRDHRVHHKYSETD-----IGEGFHNYHHTFPFD-----RTGDGSA---	[294aa]
Nematode	fat-6-Worm	-YKMEIVWRN-----GITAGAHRLWSHKSYKAT-----EWARDHRCHHKWTDTD-----VGEGGHNFHHTFPQD-----NHGCDIQRGK	[339aa]
Nematode	fat-7-Worm	-YKMEIVWRN-----GITAGAHRLWSHKSYKAT-----EWARDHRCHHKWTDTD-----VGEGGHNFHHTFPQD-----NHGSEESRKK	[338aa]
Nematode	fat-5-Worm	-IKMEIVWKN-----GVTGGAHRLWAHRAYKAT-----DWARDHRCHHKWTDTD-----VGEGGHNYHHTFPQD-----KFGCETEREK	[333aa]
Drosophila	Desat1-Fruit_fly	KRRLKLVWRN-----GITAGAHRLWAHRSYKAK-----HWARDHRVHHKYSETD-----FGEGWHNYHHVFPWD-----RTGDGTHATW	[383aa]
Drosophila	Desat2-Fruit_fly	KRRLPLVWRN-----GVTAGAHRLWAHRTYKAK-----HWARDHRVHHKYSETD-----FGEGWHNYHHAFPWD-----RTGDGSHELW	[361aa]
Drosophila	Fad2-Fruit_fly	GSKLELVWVN-----GVSGGAHRLWAHRTFKAN-----YWARDHRVHHKYTETD-----VGEGYHNYHHVFPWD-----RTGDGSHELW	[355aa]
Amphibian	scd-Tropical clawed frog	KPPIKLVWRN-----GVTAGAHRLWSHRSYKAK-----YEWARDHRVHHKYSET-----IGEGFHNYHHTFPFD-----RTGDGSHRSG	[338aa]
Freshwater economic fish	scd-Zebrafish	KPPMKIVWRN-----GITAGVHRLWSHRSYKAT-----EWSRDHRVHHKYSETD-----IGEGYHNYHHTFPYD-----RTGDGSYRSG	[326aa]
Freshwater economic fish	scdb-Zebrafish	SPPVQIVWRN-----GITAGAHRLWSHRSYRAS-----EWARDHRVHHKFSETD-----IGEGFHNYHHTFPHD-----RTGDGSHKSG	[316aa]
Freshwater economic fish	scd-Carp	KPPTVIVWRN-----------------------------EWSRDHRVHHKYSETD-----IGEGFHNYHHTFPFD-----RTGDGSHWSG	[325aa]
Freshwater economic fish	scd-Goldfish	KPPIVIVWRN-----GITAGAHRLWSHRSYKAS-----EWSRDHRVHHKYSETD-----IGEGFHNYHHTFPFD-----RTGDGSHWSG	[326aa]
Freshwater economic fish	scdb-Goldfish	SPPVKIVWRN-----GITAGAHRLWSHRSYKAS-----EWARDHRVHHKFSETQ-----IGEGFHNYHHTFPHD-----RTGDGSHKSG	[378aa]
Freshwater economic fish	delta-9-Grass carp	KPPIVVVWRN-----GITAGAHRLWSHRSYKAS-----EWSRDHRVHHKYSETD-----IGEGFHNYHHTFPFD-----RTGDGSHRSG	[324aa]
Freshwater economic fish	scd-Channel catfish	EAPMVVVWRN-----GITAGAHRLWSHRSYKAS-----EWSRDHRVHHKYSETD-----IGEGFHNYHHTFPYD-----RTGDGSHRTG	[330aa]
Freshwater economic fish	scdb-Channel catfish	SPPLKIVWRN-----GVTAGAHRLWSHRSYKAK-----EWARDHRVHHKYSETH-----VGEGFHNYHHTFPYD-----RTGDGSHKSG	[331aa]
Freshwater economic fish	SCD-Yellow catfish	DPPVVVVWRN-----GITAGAHRLWSHRSYKAS-----EWVRDHRVHHKYSETD-----IGEGFHNYHHTFPYD-----RTGDGSHRSG	[333aa]
Freshwater economic fish	scd-Tilapia	KPPRIIVWKN-----GVTAGAHRLWSHRSYKAT-----EWARDHRVHHKYSETD-----IGEGFHNYHHTFPYD-----RTGDGSHRSG	[335aa]
Freshwater economic fish	scd-Eel	KPPVIIVWKN-----GITAGAHRLWSHRSYKAS-----EWARDHRVHHKYSETD-----IGEGFHNYHHTFPYD-----RTGDGSHRSG	[333aa]
Freshwater economic fish	scdb-Bass	KPPKMMVWRN-----GVTAGAHRLWSHRSYKAS-----EWARDHRVHHKYSETD-----IGEGFHNYHHTFPFD-----RTGDGSHKSG	[334aa]
Euryhaline fish	scdb-Rainbow trout	KPPMRLVWRN-----GITAGAHRLWSHKSYKAS-----EWARDHRVHHKYSETD-----IGEGFHNYHHTFPFD-----RTGDGSHKTG	[330aa]
Euryhaline fish	SCD5-Rainbow trout	---LGIVWRN-----GVTAGAHRLWSHRSYRAK-----EWARDHRVHHKFSETD-----IGEGFHNFHHTFPFD-----RTGDGSYGAV	[331aa]
Euryhaline fish	ACOD-Salmon	KPPMRLVWRN-----GITAGAHRLWSHKSYKAS-----EWARDHRVHHKYSETD-----IGEGFHNYHHTFPFD-----RTGDGSHKTG	[331aa]
Euryhaline fish	scdb-Salmon	KPRMRLVWRN-----GITAGAHRLWSHKSYKAS-----EWARDHRVHHKYSETD-----IGEGFHNYHHTFPFD-----RTGDGSHKTG	[331aa]
Euryhaline fish	SCD5-Salmon	---LGIVWRN-----GVTAGAHRLWSHRSYRAK-----EWARDHRVHHKFSETD-----IGEGFHNFHHTFPFD-----RTGDGSHKTG	[342aa]
Marine economic fish	SCD1-Large yellow croaker	KPPRMLVWRN-----GVTAGAHRLWSHRSYKAS-----EWARDHRVHHKYSETD-----IGEGFHNYHHTFPFD-----RTGDGSYKSG	[335aa]
Marine economic fish	scd-Fugu	KPAKIIVWKN-----GITAGAHRLWSHRTYKAS-----EWARDHRVHHKYSETD-----TGEGFHNYHHSFPYD-----RTGDGSHRSG	[333aa]
Marine economic fish	scdb-Fugu	KPPRRLVWRN-----GVTAGAHRLWSHRSYKAS-----EWARDHRVHHKFSETD-----IGEGFHNYHHTFPFD-----RTGDGSHRSG	[371aa]
Marine economic fish	SCD1a-Snapper	KPGTIIVWKN-----GVTAGAHRLWSHRSYKAS-----EWARDHRVHHKYSETD-----IGEGFHNYHHSFPYD-----RTGDGSHRSG	[335aa]
Marine economic fish	scdb-Snapper	KPPRTLVWRN-----GVTAGAHRLWSHRSYKAS-----EWARDHRVHHKYSETD-----IGEGFHNYHHTFPFD-----RTGDGSYKSG	[335aa]
Marine economic fish	scd-Cod	KPPKEIVWKN-----GVTAGAHRLWSHRTYKAS-----EWARDHRVHHKYSETD-----IGEGFHNYHHTFPYD-----RTGDASIRSG	[334aa]
Marine economic fish	scdb-Cod	KPPRRLVWRN-----GVTAGAHRLWSHRTYKAS-----EWARDHRTHHKYSETD-----VGEGFHNYHHTFPFD-----RTGDASNKSG	[329aa]
Chondrichthian	scd5-Shark	---FIIVWRN-----GVTAGAHRLWSHRSYTAK-----EWSRDHRVHHKYSETD-----IGEGFHNYHHTFPFD-----RTGDKSA---	[325aa]
Chondrichthian	scd-Shark	KPPMRLVWRN-----GITAGAHRLWSHRSYRAT-----EWARDHRVHHKFSETD-----IGEGFHNYHHTFPYD-----RTGDRSVRSG	[360aa]
	scd5-Amphioxus	APPLQIVWRN-----GVTAGAHRLWAHRSYKAR-----DWSRDHRVHHKYSETP-----MGEGWHNYHHTFPYD-----RTGDGTWKNS	[328aa]
	acod-Ciona	SFLHQIQWFN-----GVTAGVHRYWCHRSYKAT-----NWATNHRVHHKFSETD-----MGEGYHNYHHVFPHD-----KNLLRKEQ--	[372aa]
