# Supplementary material for: Genome‐wide comparative identification and analysis of membrane‐FADS‐like superfamily genes in freshwater economic fishes
Source: FEBS Open Bio. 2023 Mar 16;13(6):1067–85. doi: 10.1002/2211-5463.13594 (PMC10240347; doi:10.1002/2211-5463.13594)
Supplement: Supplementary file 3 — Fig. S3. The DEGS family protein multiple sequence alignment and conserved His motif. [file FEB4-13-1067-s009.pdf]

Group	Sequence name	1 – 10	81 – 89 – 93 – 99	123 – 128 – 132 – 138	254 – 259 – 263 – 269	314 – 322	Length
Mammal	DEGS1-Human	MGSRVSREDF-----	NHSMTLAI**HEIAH**NAAFGN-----	SFKRY**HMDHH**RYLGAD-----	FNVGY**HNEHH**DFPNIP-----	RHQKGEMVL	[323aa]
	DEGS2-Human	MGNSASRSDF-----	NHSLTLAI**HDISH**NAAFGT-----	SFKKY**HVDHH**RYLGGD-----	FNVGY**HVEHH**DFPSIP-----	RVYRLAKDG	[323aa]
	Degs1-Mouse	MGSRVSREEF-----	NHSMTLAI**HEISH**NFPFGH-----	SFKRY**HMDHH**RYLGAD-----	FNVGY**HNEHH**DFPNVP-----	RPPKGNEIL	[323aa]
	Degs2-Mouse	MGNSAARSDF-----	NHSLTLAI**HDISH**NTAFGT-----	SFKKY**HVDHH**RYLGGD-----	FNVGY**HMEHH**DFPSIP-----	RKCKLAKDH	[323aa]
	Degs2-Rat	MGNSAARSDF-----	NHSLTLAI**HDISH**NTAFGT-----	SFKKY**HVDHH**RYLGGD-----	FNVGY**HVEHH**DFPSIP-----	RKCKLAKDQ	[323aa]
	Degs1-Rat	MGNRVSREEF-----	NHSMTLAI**HEISH**NFPFGH-----	SFKRY**HMDHH**RYLGAD-----	FNVGY**HNEHH**DFPNVP-----	RPPKGNEIQ	[323aa]
Aves	DEGS1-Chicken	MGNTVAREDF-----	SHSMTLAI**HEISH**NSAFGN-----	SFKRY**HMDHH**RYLGGD-----	FNVGY**HNEHH**DFPNIP-----	RQLKGEVKQ	[322aa]
	DEGS2-Chicken	MGNRVTRGDF-----	NHSLTLAI**HDISH**NVAFGN-----	SFKKY**HIDHH**RYLGGD-----	FNVGY**HMEHH**DFPSIP-----	RKCKLAKES	[323aa]
Amphibian	degs1-Tropical clawed frog	MGNSAAREDF-----	SHSMTLAI**HEISH**NSAFGN-----	SFKRY**HMDHH**RYLGGD-----	FNVGY**HNEHH**DFPSVP-----	RKLKGDLKL	[323aa]
	degs2-Tropical clawed frog	MGNTVTRGDF-----	NHSLTLAI**HDISH**NVAFGN-----	SFKKY**HIDHH**RYLGGD-----	FNVGY**HTEHH**DFPSIP-----	RKCLLVNKS	[322aa]
**Freshwater economic fish**	degs1-Zebrafish	MGNRVAREDF-----	NHSMTLAI**HEISH**NTAFGN-----	SFKRY**HLDHH**RYLGGD-----	FNVGY**HNEHH**DFPSIP-----	RKLVGDVKQ	[323aa]
	degs2-Zebrafsh	MGKAAGRWDF-----	NHSLTLAI**HDISH**NVAFGT-----	SFKKY**HIDHH**RYLGGD-----	FNVGY**HMEHH**DFPSIP-----	RQYTLGKKE	[323aa]
	degs1-Carp	MGNRVAREDF-----	NHSMTLAI**HEISH**NTAFGN-----	SFKRY**HLDHH**RYLGGD-----	FNVGY**HNEHH**DFPSIP-----	RLTGEVKQE	[323aa]
	degs2-Carp	MGKAVGRWDF-----	NHSLTLAI**HDISH**NVAFGT-----	SFKKY**HIDHH**RYLGGD-----	FNVGY**HMEHH**DFPSIP-----	RQYKLGKKE	[322aa]
	degs1-Grass carp	MGNRVVREDF-----	NHSMTLAI**HEISH**NTAFGN-----	SFKRY**HLDHH**RYLGGD-----	FNVGY**HNEHH**DFPSIP-----	RLTGDIKQE	[323aa]
	degs2-Grass carp	MGKAVGRWDF-----	NHSLTLAI**HDISH**NVAFGT-----	SFKKY**HIDHH**RYLGGD-----	FNVGY**HMEHH**DFPSIP-----	RQYKLGKKE	[322aa]
	degs1-Goldfish	MGNRVAREDF-----	NHSMTLAI**HEISH**NTAFGN-----	SFKRY**HLDHH**RYLGGD-----	FNVGY**HNEHH**DFPSIP-----	RLTGEVKQE	[323aa]
	degs2-Goldfish	MGKAVGRWDF-----	NHSLTLAI**HDISH**NVAFGT-----	SFKKY**HIDHH**RYLGGD-----	FNVGY**HMEHH**DFPSIP-----	RRYELGKNE	[322aa]
	degs1-Channel catfish	MGNRVAREDF-----	NHSMTLAI**HEISH**NTAFGN-----	SFKRY**HLDHH**RYLGGD-----	FNVGY**HNEHH**DFPSIP-----	KLKGDLKLE	[323aa]
	degs2-Channel catfish	MGKAGERVDF-----	NHSLTLAI**HDISH**NIAFGN-----	SFKKY**HIDHH**RYLGGD-----	FNVGY**HMEHH**DFPGIP-----	REYKLAKQE	[305aa]
	degs1-Yellow catfish	MGNRVARDDF-----	NHSMTLAI**HEISH**NTAFGN-----	SFKRY**HLDHH**RYLGGD-----	FNVGY**HNEHH**DFPSIP-----	KLKGDLKLE	[323aa]
	degs2-Yellow catfish	MGKAGVRGDF-----	NHSLTLAI**HDISH**NIAFGN-----	SFKKY**HIDHH**RYLGGD-----	FNVGY**HMEHH**DFPSIP-----	REYKLAKQE	[322aa]
	degs1-Tilapia	MGNRVAREDY-----	NHSMTLAI**HEISH**NTAFGN-----	SFKRY**HLDHH**RYLGGD-----	FNVGY**HNEHH**DFPSIP-----	RKLKGEVKQ	[323aa]
	degs2-Tilapia	MGKTGGRGDF-----	NHSLTLAI**HDISH**NVAFGN-----	AFKKY**HIDHH**RYLGGD-----	FNVGY**HMEHH**DFPSIP-----	REYKLSKQE	[322aa]
	degs1-Eel	MGNRVAREDF-----	NHSMTLAI**HEISH**NAAFGN-----	SFKRY**HLDHH**RYLGGD-----	FNVGY**HNEHH**DFPSIP-----	RKLKGEFKL	[323aa]
	degs2-Eel	MGKKGGRADF-----	NHSLTLAI**HDISH**NVAFGN-----	SFKKY**HIDHH**RYLGGD-----	FNVGY**HMEHH**DFPSIP-----	REYKLSKQG	[323aa]
	degs1-Bass	MGNRVAREDY-----	NHSMTLAI**HEISH**NTAFGN-----	SFKRY**HLDHH**RYLGGD-----	FNVGY**HNEHH**DFPSIP-----	KLKGEVKQE	[323aa]
	degs2-Bass	MGKTGGRHDF-----	NHSLTLAI**HDISH**NVAFGN-----	SFKKY**HIDHH**RYLGGD-----	FNVGY**HMEHH**DFPSIP-----	REYKLSKQE	[322aa]
Euryhaline fish	degs1-Rainbow trout	MGNRVPREDY-----	NHSMTLAI**HEISH**NTAFGN-----	SFKRY**HLDHH**RYLGGD-----	FNVGY**HNEHH**DFPSIP-----	RRLKGDVKL	[323aa]
	degs2-Rainbow trout	MDKTGERGDF-----	NHSLTLAI**HDISH**NVAFGN-----	SFKKY**HIDHH**RYLGGD-----	FNVGY**HMEHH**DFPSIP-----	REYKLVKQE	[322aa]
	degs1-Salmon	MGNRVAREDY-----	NHSMTLAI**HEISH**NTAFGN-----	SFKRY**HLDHH**RYLGGD-----	FNVGY**HNEHH**DFPSIP-----	RRLKGDIKQ	[323aa]
	degs2-Salmon	MDKTGERGDF-----	NHSLTLAI**HDISH**NVAFGN-----	SFKKY**HIDHH**RYLGGD-----	FNVGY**HMEHH**DFPSIP-----	REYKLVKQE	[327aa]
Marine economic fish	degs1-Large yellow croaker	MGNQVAREDF-----	NHSMTLAI**HEVSH**NTAFGN-----	SFKRY**HLDHH**RYLGGD-----	FNVGY**HNEHH**DFPSIP-----	RRLKGDVKQ	[323aa]
	degs2-Large yellow croaker	MGKTGGRDDF-----	NHSLTLAI**HDISH**NVAFGN-----	SFKKY**HVDHH**RYLGGD-----	FNVGY**HMEHH**DFPSIP-----	RQYKLNKQG	[391aa]
	degs1-Turbot	MGNRVAREDY-----	NHSMTLAI**HEISH**NTAFGN-----	SFKRY**HLDHH**RYLGGD-----	FNVGY**HNEHH**DFPSIP-----	KLKGDVKQE	[323aa]
	degs2-Turbot	MGKTGGRGDF-----	NHSLTLAI**HDISH**NVAFGN-----	SFKKY**HIDHH**RYLGGD-----	FNVGY**HMEHH**DFPSIP-----	REYKLSKQE	[322aa]
	degs1-Fugu	MGNRVAREDY-----	NHSMTLAI**HEVSH**NTAFGN-----	SFKRY**HLDHH**RYLGGD-----	FNVGY**HNEHH**DFPSIP-----	RKLKGDVKQ	[323aa]
	degs2-Fugu	MGKASGRHDF-----	NHSLTLAI**HDISH**NVAFGN-----	AFKKY**HIDHH**RYLGGD-----	FNVGY**HMEHH**DFPSIP-----	RKYKLSKQE	[322aa]
	degs1-Snapper	MGNRVAREDY-----	NHSMTLAI**HEVSH**NTAFGN-----	SFKRY**HLDHH**RYLGGD-----	FNVGY**HNEHH**DFPSIP-----	RKLKGDVKQ	[323aa]
	degs2-Snapper	MGKTGGRDDF-----	NHSLTLAI**HDISH**NVAFGN-----	SFKKY**HVDHH**RYLGGD-----	FNVGY**HMEHH**DFPSIP-----	RTYKISKKE	[322aa]
	degs1-Cod	MGNRVVREDY-----	NHSMTLAI**HEISH**NTAFGN-----	SFKRY**HLDHH**RYLGGD-----	FNVGY**HNEHH**DFPSVP-----	RTLKGDVKQ	[323aa]
	degs2-Cod	MGKTGGRGDF-----	NHSLTLAI**HDISH**NVAFGN-----	SFKKY**HIDHH**RYLGGD-----	FNVGY**HMEHH**DFPSIP-----	REYKIVKQG	[322aa]
chondrichthian	degs1-Shark	MGNRVARDDY-----	SHSMTLAI**HEIAH**NAAFGN-----	SFKRY**HMDHH**RYMGGH-----	FNVGY**HNEHH**DFPSVP-----	RTLK-DAKL	[439aa]
	degs2-Shark	MGLKPRRDDF-----	NHSLTLAI**HDISH**NVAFGN-----	SFKKY**HIDHH**RYLGGD-----	FNVGY**HMEHH**DFPSIP-----	RECKLVKGN	[322aa]
	degs1-Ciona	MGNIVQRSSY-----	NHSLTLAV**HELSH**NRAFGH-----	SFKKY**HLEHH**RQLGVD-----	FNVGY**HMEHH**DFPSIT-----	RPHLKDTQ-	[323aa]
