# Supplementary figures and images for: Genome‐wide comparative identification and analysis of membrane‐FADS‐like superfamily genes in freshwater economic fishes
Source: FEBS Open Bio. 2023 Mar 16;13(6):1067–85. doi: 10.1002/2211-5463.13594 (PMC10240347; doi:10.1002/2211-5463.13594)

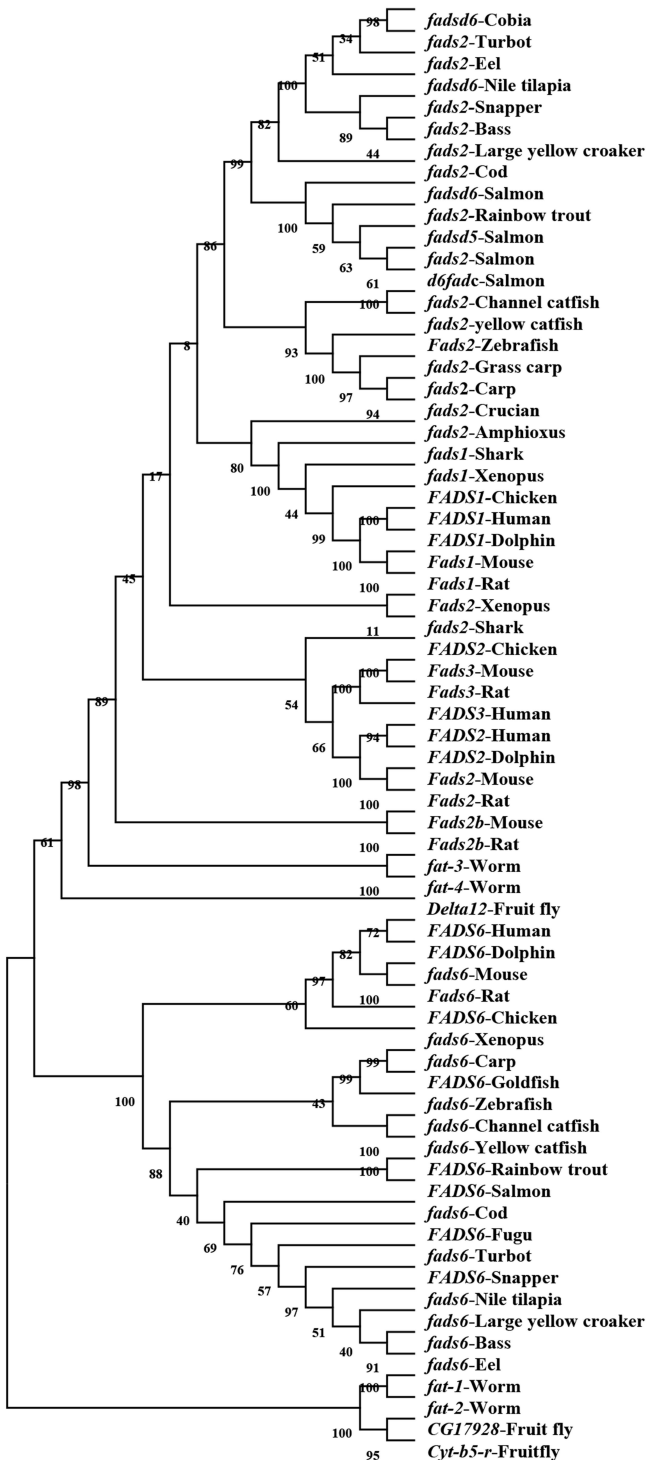

Supplement: Supplementary file 4 — Fig. S4. NJ tree of the FADS gene family. [file FEB4-13-1067-s007.pdf]

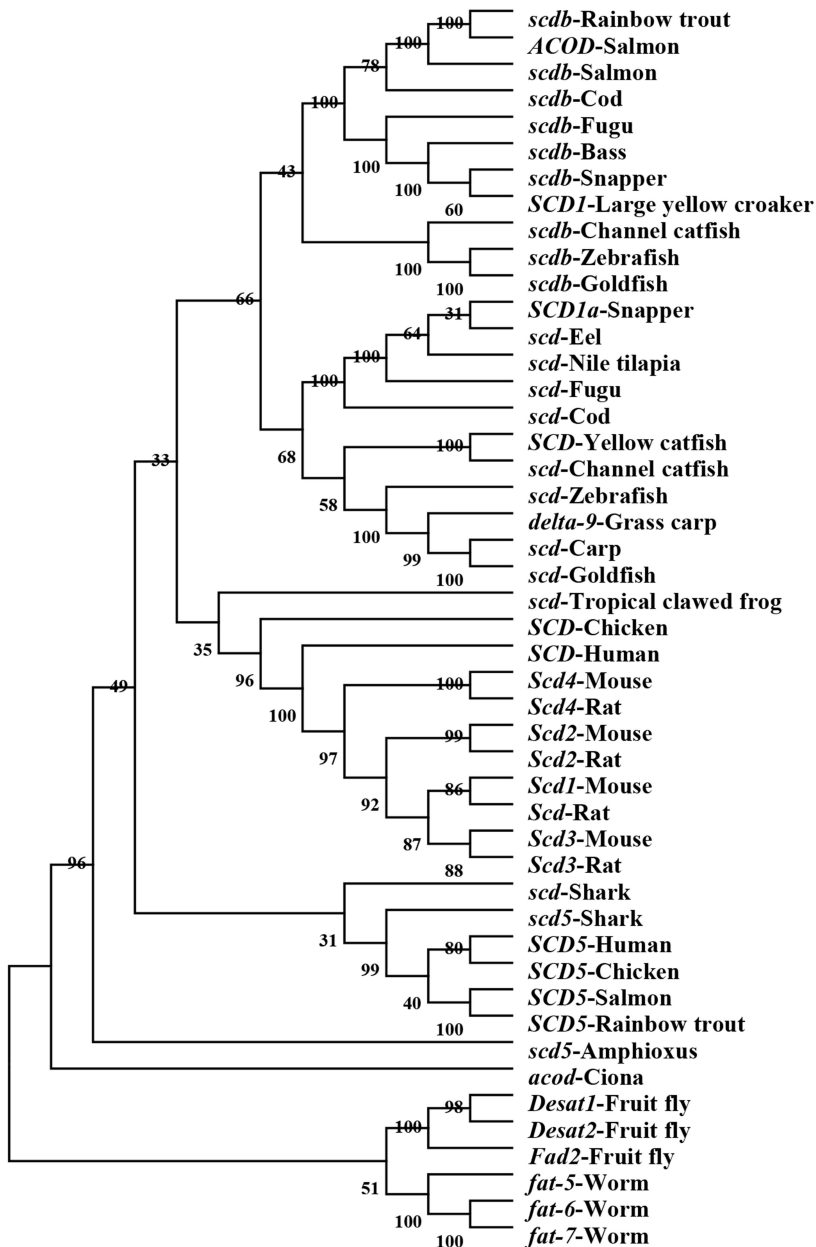

Supplement: Supplementary file 5 — Fig. S5. NJ of the SCD gene family. [file FEB4-13-1067-s012.pdf]

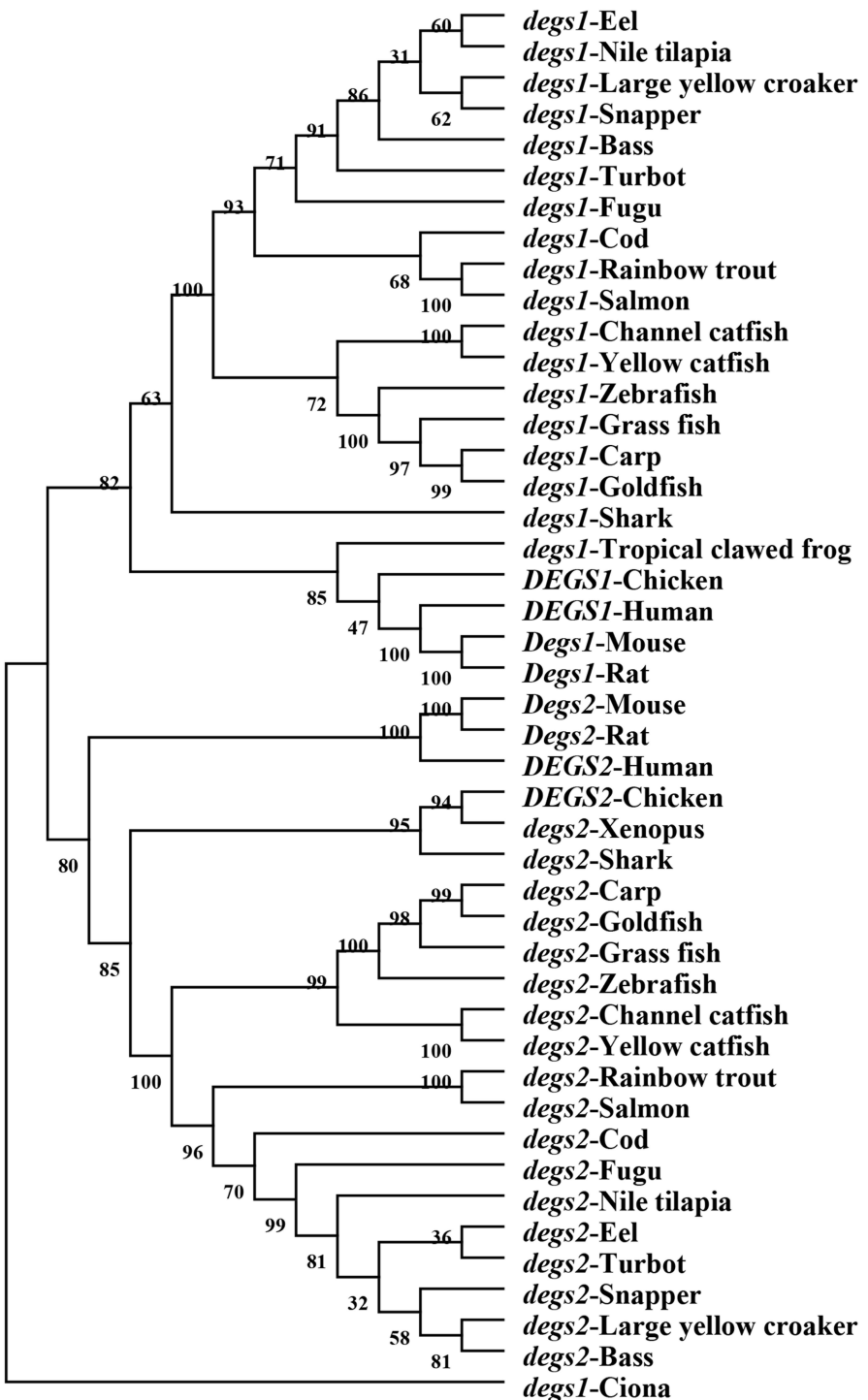

Supplement: Supplementary file 6 — Fig. S6. NJ tree of the DEGS gene family. [file FEB4-13-1067-s001.pdf]

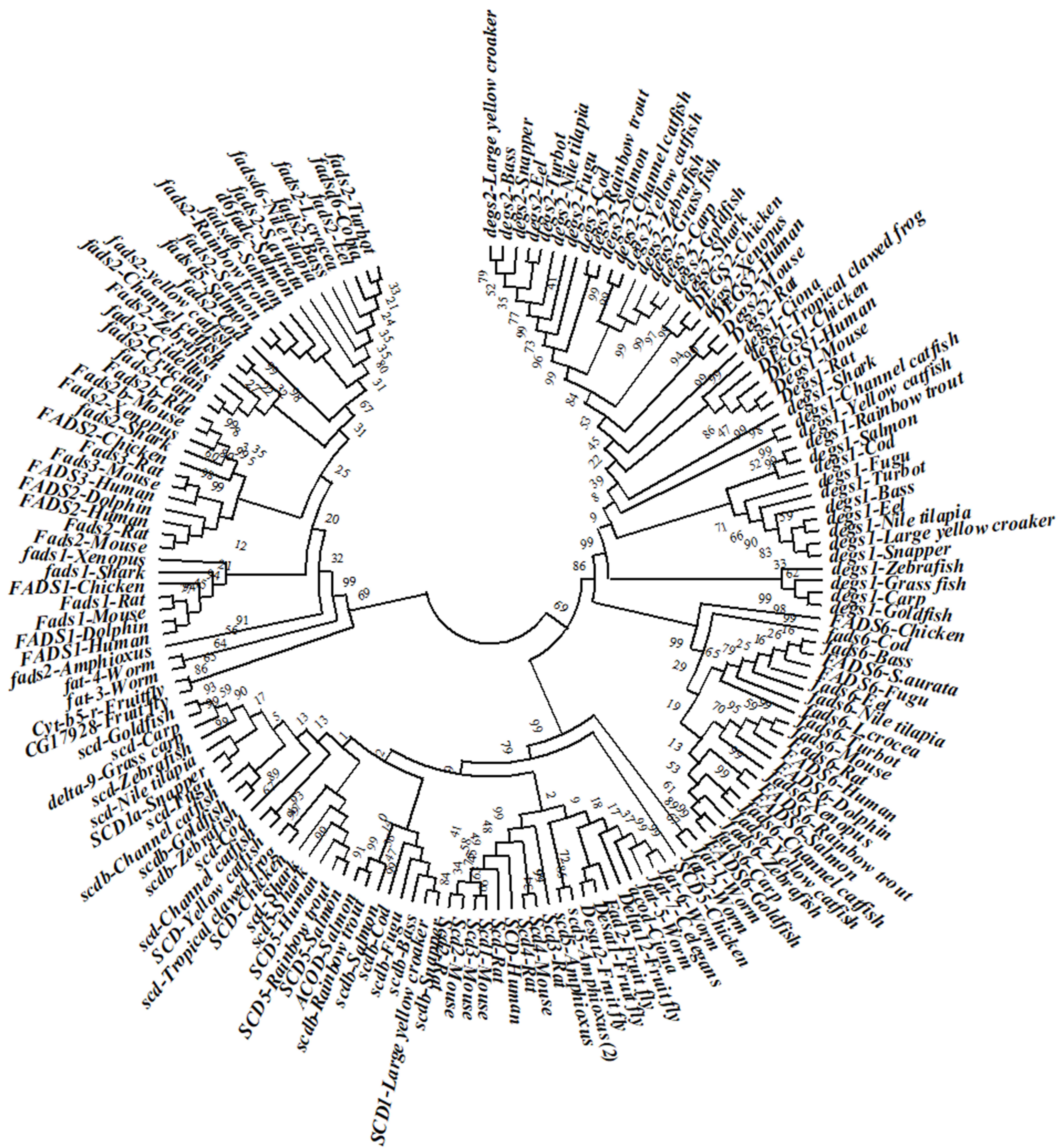

Supplement: Supplementary file 7 — Fig. S7. NJ tree of the FADSs gene superfamily. [file FEB4-13-1067-s006.pdf]

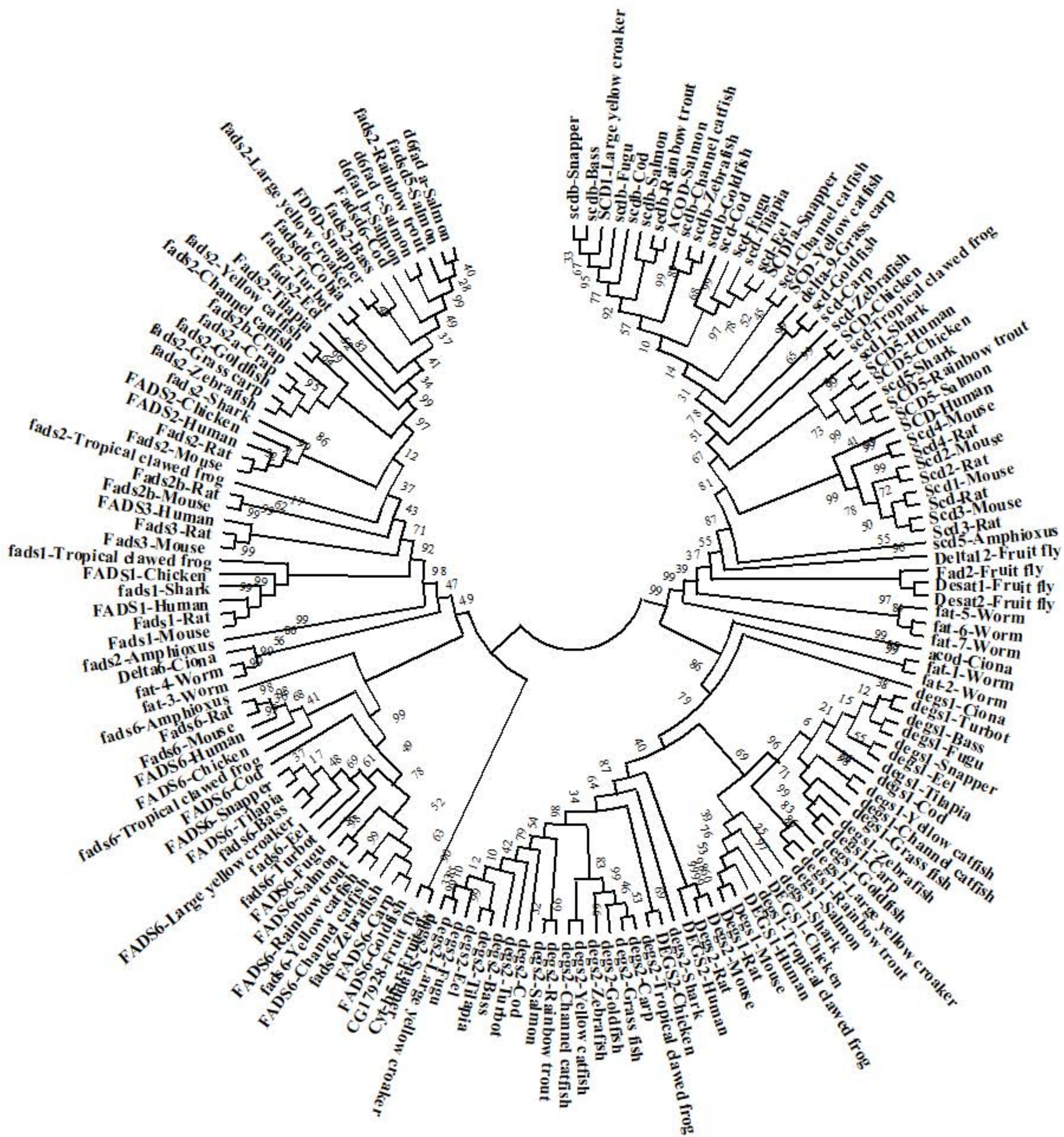

Supplement: Supplementary file 8 — Fig. S8. ML tree of FADSs superfamily proteins. [file FEB4-13-1067-s008.pdf]

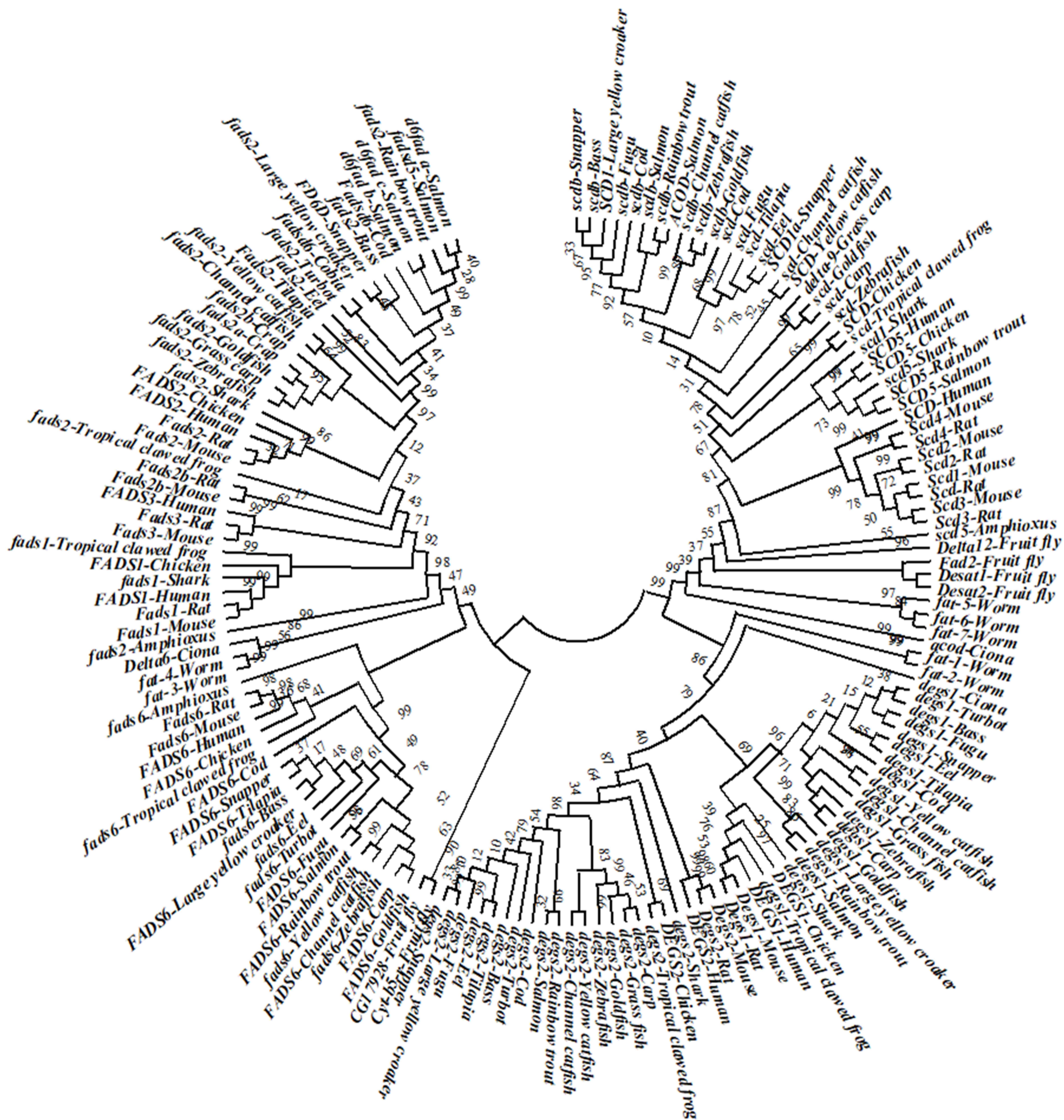

Supplement: Supplementary file 9 — Fig. S9. ML tree of FADSs superfamily genes. [file FEB4-13-1067-s014.pdf]
