# Supplementary material for: Genome‐wide comparative identification and analysis of membrane‐FADS‐like superfamily genes in freshwater economic fishes
Source: FEBS Open Bio. 2023 Mar 16;13(6):1067–85. doi: 10.1002/2211-5463.13594 (PMC10240347; doi:10.1002/2211-5463.13594)
Supplement: Supplementary file 11 — Table S2. The atypical structure of protein‐coding genes. Gold shading indicates important freshwater commercial fish. [file FEB4-13-1067-s004.docx]

|  | **Orgnism-types** | **Transcript ID** | **Exson** | **Intron** |
| --- | --- | --- | --- | --- |
| **FADS1 -FADS3 (12+11)** | **Shark-fads1** | **ENSCMIT00000027181.1** | **11** | **10** |
|  | **Salmon-fadsd5** | **ENSSSAT00000131168.1** | **14** | **13** |
|  | **Rat-Fads2** | **ENSRNOT00000027756.6** | **10** | **9** |
|  | **Xenopus-fads2** | **ENSXETT00000088032.1** | **14** | **13** |
|  | **Zebrafish-fads2** | **ENSDART00000023278.7** | **13** | **12** |
|  | **Salmon-fads2** | **ENSSSAT00000131319.1** | **8** | **7** |
|  | **Carp-fads2** | **ENSCCRT00000051501.1** | **11** | **10** |
|  | **Channel catfhish-fads2** | **ENSIPUT00000013435.1** | **13** | **12** |
|  | **Large yellow croaker-fads2** | **ENSLCRT00005018010.1** | **13** | **12** |
|  | **Salmon-fadsd6** | **ENSSSAT00000104105.1** | **13** | **12** |
|  | **Cod-Fadsd6** | **ENSGMOT00000049303.1** | **13** | **12** |
|  | **Snapper-FD6D** | **ENSSAUT00010040776.1** | **13** | **12** |
|  | **Salmon-d6fadc** | **ENSSSAT00000127699.1** | **13** | **12** |
|  | **Ciona-delta6** | **ENSCINT00000019998.3** | **6** | **5** |
| **FADS6 (6+5)** | **Chicken-FADS6** | **ENSGALT00000059705.2** | **7** | **6** |
|  | **Xenopus-fads6** | **ENSXETT00000014993.4** | **7** | **6** |
|  | **Zebrafish-fads6** | **ENSDART00000189164.1** | **7** | **6** |
|  | **Salmon-FADS6** | **ENSSSAT00000005574.1** | **7** | **6** |
|  | **Carp-FADS6** | **ENSCCRT00000064633.1** | **7** | **6** |
|  | **Cod-FADS6** | **ENSGMOT00000011770.2** | **7** | **6** |
|  | **Swamp Eel-fads6** | **ENSMALT00000026000.1** | **8** | **7** |
|  | **Tilapia-FADS6** | **ENSONIT00000067017.1** | **7** | **6** |
| **SCD (6+5)** | **Shark-scd** | **ENSCMIT00000022379.1** | **8** | **7** |
|  | **Carp-scd** | **ENSCCRT00000025936.1** | **8** | **7** |
|  | **Cod-scd** | **ENSGMOT00000043419.1** | **5** | **4** |
|  | **Channel catfhish-scd** | **ENSIPUT00000002020.1** | **7** | **6** |
|  | **Swamp Eel-scd** | **ENSMALT00000002718.1** | **5** | **4** |
|  | **Human-SCD5** | **ENST00000319540.9** | **5** | **4** |
|  | **Chicken-SCD5** | **ENSGALT00000087886.2** | **5** | **4** |
|  | **Shark-scd5** | **ENSCMIT00000018790.1** | **5** | **4** |
|  | **Rainbow trout-SCD5** | **ENSOMYT00000042403.1** | **5** | **4** |
|  | **Worm-fat-5** | **W06D12.3.1** | **3** | **2** |
|  | **Worm-fat-6** | **VZK822L.1a.2** | **5** | **4** |
|  | **Worm-fat-7** | **F10D2.9.1** | **5** | **4** |
|  | **Cod-scdb** | **ENSGMOT00000013805.2** | **5** | **4** |
|  | **Rainbow trout-scdb** | **ENSOMYT00000114873.1** | **5** | **4** |
| **DEGS (3+2)** | **Channel catfhish-degs2** | **ENSIPUT00000032603.1** | **2** | **1** |

**Table S2 The atypical structure of some protein-coding genes**
