# Supplementary material for: Genome‐wide comparative identification and analysis of membrane‐FADS‐like superfamily genes in freshwater economic fishes
Source: FEBS Open Bio. 2023 Mar 16;13(6):1067–85. doi: 10.1002/2211-5463.13594 (PMC10240347; doi:10.1002/2211-5463.13594)
Supplement: Supplementary file 13 — Table S4. List of sequence IDs used for constructing the phylogenetic tree of FADSs superfamily proteins. [file FEB4-13-1067-s011.docx]

**Table S 4 List of sequence IDs used in NJ phylogenetic trees**

| Species | Accession Number | Name |
| --- | --- | --- |
| *Homo sapiens* | AAF29378.1 | FADS1 |
| Human | NP_004256.1 | FADS2 |
|  | NP_068373.1 | FADS3 |
|  | NP_001032671.2 | SCD5 |
|  | NP_005054.3 | SCD |
|  | NP_835229.3 | FADS6 |
|  | NP_003667.1 | DEGS1 |
|  | NP_996801.2 | DEGS2 |
| *Mus musculus* | NP_666206.1 | Fads1 |
| Mouse | NP_062673.1 | Fads2 |
|  | NP_001075133.1 | Fads2b |
|  | NP_068690.3 | Fads3 |
|  | NP_033153.2 | Scd1 |
|  | NP_033154.2 | Scd2 |
|  | NP_077770.1 | Scd3 |
|  | NP_899039.2 | Scd4 |
|  | NP_828874.3 | Fads6 |
|  | NP_031879.1 | Degs1 |
|  | NP_081575.2 | Degs2 |
| *Rattus norvegicus* | AAG35068.1 | Fads1 |
| Rat | BAA75496.1 | Fads2 |
|  | XP_002729233.1 | Fads2b |
|  | NP_775160.1 | Fads3 |
|  | NP_631931.2 | Scd |
|  | NP_114029.1 | Scd2 |
|  | XP_008758709.3 | Scd3 |
|  | XP_574671.7 | Scd4 |
|  | NP_001100534.1 | Fads6 |
|  | NP_445775.2 | Degs1 |
|  | NP_001017457.1 | Degs2 |
| *Danio rerio* | NP_571720.2 | fads2 |
| Zebrafish | NP_001018541.1 | scdb |
|  | NP_942110.2 | scd |
|  | XP_003199708.1 | fads6 |
|  | NP_997865.1 | degs1 |
|  | NP_001156838.1 | degs2 |
| *Gallus gallus* | XP_421052.4 | FADS1 |
| Chicken | NP_001153900.1 | FADS2 |
|  | XP_015131966.1 | SCD5 |
|  | NP_990221.1 | SCD |
|  | XP_426241.2 | FADS6 |
|  | NP_001012583.1 | DEGS1 |
|  | XP_421364.2 | DEGS2 |
| *Xenopus tropicalis* | XP_002943012.2 | fads1 |
| Tropical clawed frog | NP_001120262.1 | fads2 |
|  | XP_012808654.1 | scd |
|  | XP_012822130.1 | fads6 |
|  | NP_001007485.1 | degs1 |
|  | XP_002936627.1 | degs2 |
| *Branchiostoma belcheri* | XP_019616342.1 | fads2 |
| Amphioxus | XP_019635286.1 | scd5 |
|  | XP_019636617.1 | fads6 |
| *Ciona intestinalis* | XP_002131801.1 | Delta6 |
| Ciona | XP_002126680.2 | acod |
|  | XP_026690835.1 | degs1 |
| *Callorhinchus milii* | XP_007885635.1 | fads1 |
| Shark | XP_007885636.1 | fads2 |
|  | XP_007887154.1 | scd5 |
|  | XP_007897686.1 | scd |
|  | NP_001279205.1 | degs1 |
|  | XP_007886521.1 | degs2 |
| *Cyprinus carpio* | QEN96526.1 | fads2a |
| Carp | QEN96527.1 | fads2b |
|  | XP_018961868.1 | scd |
|  | XP_018953792.1 | fads6 |
|  | XP_042592224.1 | degs1 |
|  | XP_042598437.1 | degs2 |
| *Ctenopharyngodon idella* | AAS89346.1 | fads2 |
| Grass Carp | CAB53008.1 | delta9 |
|  | GEUQ01062843.1 | degs1 |
|  | GEUQ01036533.1 | degs2 |
| *Carassius auratus* | XP_026094349.1 | fads2 |
| Goldfish | XP_026117312 | scdb |
|  | XP_026131936 | scd |
|  | XP_026131844.1 | FADS6 |
|  | XP_026080659.1 | degs1 |
|  | XP_026141964.1 | degs2 |
| *Oncorhynchus mykiss* | AAK26745.1 | fads2 |
| Rainbow trout | XP_021469290.1 | scdb |
|  | XP_021458623.2 | SCD5 |
|  | XP_021436771.2 | FADS6 |
|  | XP_021466074.1 | degs1 |
|  | XP_021430498.1 | degs2 |
| *Ictalurus punctatus* | XP_017341187.1 | fads2 |
| Channel catfish | XP_017320070.1 | scdb |
|  | XP_017338153.1 | scd |
|  | XP_017338193.1 | FADS6 |
|  | XP_017317012.1 | degs1 |
|  | NP_001187333.1 | degs2 |
| *Tachysurus fulvidraco* | XP_027029136.1 | fads2 |
| Yellow catfish | XP_026992014.1 | SCD |
|  | XP_026989749.1 | fads6 |
|  | XP_027030307.1 | degs1 |
|  | XP_027028203.1 | degs2 |
| *Oreochromis niloticus* | NP_001266552.1 | Fads2 |
| Tilapia | XP_005471439.1 | scd |
|  | XP_025765517.1 | FADS6 |
|  | XP_003456709.1 | degs1 |
|  | XP_003453080.1 | degs2 |
| *Monopterus albus* | XP_020475029.1 | fads2 |
| Eel | XP_020460891.1 | scd |
|  | XP_020460422.1 | fads6 |
|  | XP_020463242.1 | degs1 |
|  | XP_020446493.1 | degs2 |
| *Salmo salar* | AAL82631.2 | fadsd5 |
| Salmon | AAU47273.1 | d6fad_a |
|  | ADB56961.1 | d6fad_b |
|  | NP_001165251.1 | d6fad_c |
|  | XP_014061917.1 | scdb |
|  | XP_014010587.1 | ACOD |
|  | XP_013982607.1 | SCD5 |
|  | XP_014067950.1 | FADS6 |
|  | XP_014058579.1 | degs1 |
|  | XP_014065160.1 | degs2 |
| *Micropterus salmoides* | XP_038562389.1 | fads2 |
| Bass | XP_038593057.1 | scdb |
|  | XP_038591706.1 | fads6 |
|  | XP_038593161.1 | degs1 |
|  | XP_038585026.1 | degs2 |
| *Larimichthys crocea* | NP_001290292.1 | fads2 |
| Large yellow croaker | XP_010743587.1 | SCD1 |
|  | XP_010740675.2 | FADS6 |
|  | XP_010753763.1 | degs1 |
|  | XP_010732451.1 | degs2 |
| *Scophthalmus maximus* | XP_035497335.1 | fads2 |
| Turbot | XP_035468074.1 | fads6 |
|  | XP_035462931.1 | degs1 |
|  | XP_035467271.1 | degs2 |
| *Takifugu rubripes* | NP_001072046.1 | scdb |
| Fugu | NP_001072045.1 | scd |
|  | XP_003961115.3 | FADS6 |
|  | XP_029688337.1 | degs1 |
|  | XP_003962526.1 | degs2 |
| *Sparus aurata* | XP_030281225.1 | FD6D |
| Snapper | XP_030297377.1 | scdb |
|  | XP_030256516.1 | SCD1a |
|  | XP_030256488.1 | FADS6 |
|  | XP_030297019.1 | degs1 |
|  | XP_030247236.1 | degs2 |
| *Rachycentron canadum* | ACJ65149.1 | fadsd6 |
| Cobia |  |  |
| *Gadus morhua* | XP_030222643.1 | Fadsd6 |
| Cod | XP_030234788.1 | scdb |
|  | XP_030196200.1 | scd |
|  | XP_030195240.1 | FADS6 |
|  | XP_030235813.1 | degs1 |
|  | XP_030212594.1 | degs2 |
| \| *Caenorhabditis elegans* \| \| --- \| \| Worm \| | NP_001023560.1 | fat-1 |
|  | NP_502560.1 | fat-2 |
|  | NP_001255423.1 | fat-3 |
|  | NP_001255426.1 | fat-4 |
|  | NP_507482.1 | fat-5 |
|  | NP_001255595.1 | fat-6 |
|  | NP_504814.1 | fat-7 |
| *Drosophila melanogaster* | NP_609810.1 | CG17928 |
| Fruit fly | NP_477154.1 | Cyt-b5-r |
|  | NP_652731.1 | Desat1 |
|  | NP_650201.1 | Desat2 |
|  | NP_651966.2 | Fad2 |
|  | XP_022211055.2 | acyl-CoA Delta12-desaturase |
